# Functional brain network organization during the 40-Hz auditory steady-state response in children with and without autism spectrum disorder

**DOI:** 10.3389/fpsyt.2026.1804124

**Published:** 2026-06-09

**Authors:** Mai Yasumoto, Yoshiaki Miyagishi, Tetsu Hirosawa, Yasuki Ono, Daiki Soma, Yoko Osaka, Masafumi Kameya, Keigo Yuasa, Masuhiko Sano, Ryota Takeuchi, Ye Niu, Yuko Yoshimura, Yuka Shiota, Sanae Tanaka, Chiaki Hasegawa, Mitsuru Kikuchi

**Affiliations:** 1 Department of Child and Adolescent Psychiatry, Graduate School of Medical Science, Kanazawa University, Kanazawa, Japan; 2 Department of Psychiatry and Neurobiology, Graduate School of Medical Science, Kanazawa University, Kanazawa, Japan; 3 Mirainokaze Seiwa Hospital, Iwate, Japan; 4 Research Center for Child Mental Development, Kanazawa University, Kanazawa, Japan

**Keywords:** auditory steady-state response, autism spectrum disorder, graph theory, magnetoencephalography, neurodevelopment

## Abstract

**Introduction:**

The auditory steady-state response (ASSR) at 40 Hz provides a well-established probe of gamma-band neural synchronization and is thought to reflect excitation–inhibition balance in cortical microcircuits—a mechanism implicated in autism spectrum disorder (ASD). Previous studies focusing on regional ASSR measures have yielded inconsistent findings for ASD, suggesting that system-level approaches may provide complementary information about ASD-related neurophysiological alterations.

**Methods:**

We examined source-level functional brain networks during the 40-Hz ASSR in children with ASD (n = 19) and typically developing (TD) children (n = 34) aged 5–8 years using magnetoencephalography (MEG). Functional connectivity was estimated using the phase lag index (PLI) within the 30–50 Hz band, and graph-theoretical metrics—clustering coefficient (CC), characteristic path length (PL), and small-worldness (SW)—were computed from binary undirected networks. Group differences were assessed using multiple linear regression adjusting for age, sex, and cognitive ability. Associations between network measures and autistic traits, indexed by the Social Responsiveness Scale (SRS), were examined using a regression model including diagnosis and the interaction of diagnosis × graph measure.

**Results:**

The experimental paradigm elicited ASSR-related cortical activity in both groups. A significant main effect of diagnosis was observed for characteristic PL, with children with ASD exhibiting shorter PL than TD children, indicating altered network topology during 40-Hz ASSR (p = 0.0023). No group differences were found for CC or SW. In the secondary analysis, shorter PL was associated with higher SRS total T-scores in the omnibus model (p = 0.027). The diagnosis × PL interaction was not significant, indicating that this association did not differ detectably between ASD and TD groups.

**Discussion:**

These findings suggest that graph-theoretical analysis of functional brain networks during the 40-Hz ASSR may provide complementary information about ASD-related neural organization beyond conventional ASSR measures. Shorter characteristic PL in ASD, together with its association with autistic-trait severity across the full sample, points to altered large-scale network configuration during externally driven gamma-band synchronization. The results support the value of examining functional brain network topology during the 40-Hz ASSR in ASD.

## 1 Introduction

Autism spectrum disorder (ASD) is a neurodevelopmental condition characterized by persistent difficulties in social communication and interaction, along with restricted and repetitive patterns of behaviors and interests (1). Establishing a timely and accurate diagnosis of ASD can be challenging in clinical practice, as behavioral manifestations are often subtle, heterogeneous, and influenced by developmental stage. Diagnostic complexity is further increased by practical constraints during clinical assessments and frequent co-occurring conditions and states, such as anxiety or hyperactivity (2–4). Moreover, several contextual and demographic factors—including female sex, milder symptom expression, fragmented access to care, socioeconomic disadvantage, and language barriers—may lead to under-recognition or delayed diagnosis (4–7). In light of these challenges, efforts to elucidate the biological and physiological mechanisms underlying ASD have gained increasing prominence in complementing behavioral assessment and improving diagnostic precision. Neuroimaging approaches have thus become central to contemporary ASD research (8). In particular, electroencephalography (EEG) and magnetoencephalography (MEG) offer direct measures of neural activity by capturing the brain’s electrical and magnetic signals, respectively. Both modalities are noninvasive, silent, and free of ionizing radiation, rendering them particularly well-suited for studies involving children and adolescents (9, 10).

Among MEG-measurable indices, gamma-band oscillations (30–150 Hz) have garnered considerable attention in ASD research. This interest primarily stems from cellular and optogenetic studies demonstrating that these oscillations are governed by the balance between excitation and inhibition within local cortical circuits, with fast-spiking parvalbumin-expressing (FSPV) GABAergic interneurons playing a central role in their generation and synchronization (11–13). This excitation–inhibition regulation is particularly relevant in the context of ASD, as converging evidence from animal models and human studies indicates that disruptions of this balance—especially involving GABAergic signaling—represent a core neurobiological feature of the disorder (14, 15). Among measures of gamma-band oscillatory activity, the auditory steady-state response (ASSR) at 40 Hz provides a well-characterized assay of gamma-band synchronization in the human auditory cortex (16). The ASSR reflects sustained phase-locked neural activity that entrains to the temporal structure of a periodic auditory stimulus (17), with maximal amplitude observed at stimulation rates around 40 Hz in humans (18). The 40-Hz ASSR is particularly interesting in ASD research because, at the microcircuit level, it is thought to arise from interactions between pyramidal neurons and FSPV interneurons, with N-methyl-D-aspartate (NMDA) receptor–mediated excitation of FSPVcells playing a critical role in maintaining gamma-frequency entrainment (13, 19–21). Consistent with this mechanism, pharmacological manipulation of NMDA receptor function *in vitro* and *in vivo* has been shown to attenuate or enhance gamma-band oscillations in the 30–80 Hz range, including the 40-Hz ASSR (22, 23).

Early research has largely localized generators of the 40-Hz ASSR to the primary auditory cortex, most notably the superior temporal plane (24, 25) and Heschl’s gyrus (26). Thus, analyses of the 40-Hz ASSR have commonly been restricted to these auditory cortical regions. However, findings from studies examining regional ASSR measures in ASD have been inconsistent. Several studies have reported group differences between ASD and typically developing (TD) individuals (27–31). For example, Wilson et al.’s (27) early MEG study focused on children and adolescents demonstrated higher 40-Hz ASSR power in TD individuals compared with those with ASD in the left hemisphere. A subsequent study reported larger ASSR power and phase-locking factors in adult control participants than in parents of children with ASD, suggesting a possible familial effect (28). Similarly, Seymour et al. (29) observed reduced 40-Hz ASSR responses in adolescents with ASD relative to controls. By contrast, other studies have failed to detect significant group differences in regional 40-Hz ASSR measures (32–34). For instance, Edgar et al. (32) and Stroganova et al. (33) reported no significant differences in ASSR power or phase locking between TD children and adolescents and those with ASD. Consistent with these findings, in a previous study conducted by our group involving children aged 5–7 years, we found no significant group differences in ASSR measures (34). Taken together, these inconsistent results suggest that analyses focusing solely on regional ASSR measures may be insufficient to capture the neurophysiological alterations associated with ASD, underscoring the need for alternative analytical approaches.

Accumulating evidence indicates that beyond its classical localization to the primary auditory cortex, ASSR-related activity extends beyond auditory regions and engages distributed cortical networks (35–37). Responses at or near 40 Hz have been reported in frontal and subcortical structures (35), as well as in the occipital cortex, precentral gyrus, and superior parietal lobule (36), with additional involvement of distributed frontal and parietal areas noted (37). Importantly, activity across these regions does not appear to arise independently; instead, these spatially distributed neural populations exhibit coordinated engagement, suggesting organized interactions among multiple cortical and subcortical nodes (38). Furthermore, a recent study suggests that white matter bundles connecting the right frontal, parietal, and occipital cortices support the generation of the 40 Hz ASSR (39). Overall, these findings indicate that ASSR-related activity across distributed brain regions is coordinated rather than independent, supporting the involvement of large-scale neural interactions. Thus, the 40 Hz ASSR is likely to emerge from a widely distributed functional brain network rather than from isolated cortical generators.

Graph-theoretical approaches provide a powerful framework for characterizing the topological organization of functional brain networks. In studies using EEG or MEG, the brain can be parcellated into discrete regions with activity patterns that interact dynamically over time. Graph theory has been widely adopted in network neuroscience to capture and quantify the structure of such complex interregional interactions (40). Within this framework, a complex system is abstracted as a graph comprising nodes and edges, where nodes correspond to anatomically or functionally defined brain regions and edges represent functional connections between pairs of regions (41). This abstraction enables the characterization of global and local network properties using a small set of quantitative metrics. Among the various graph-theoretical measures, the mean clustering coefficient (CC) and average shortest path length (PL) are among the most fundamental, well-validated, and commonly reported indices. The CC quantifies the extent to which neighboring nodes of a given node are interconnected, thereby indexing the degree of local clustering within the network. Higher values of CC are generally interpreted as reflecting greater functional segregation, corresponding to a network’s capacity for specialized, local information processing (42). By contrast, the average shortest PL reflects the mean number of edges required to connect any two nodes in the network, averaged across all node pairs. Shorter PLs indicate more efficient global communication and are considered to reflect functional integration across distributed brain regions (42). In healthy brains, high clustering and short PLs typically coexist, forming a “small-world” configuration that optimally balances local specialization and global integration. This organization lies between highly ordered networks, characterized by high clustering and long PLs, and random networks, characterized by low clustering and short PLs (43). This intermediate topology is thought to support an efficient balance between functional segregation and integration (43). Disruptions of this balance have been reported across diverse neuropsychiatric and neurological conditions, including depression (44), dementia (45), and schizophrenia (46).

To our knowledge, no prior studies have directly compared functional brain networks during the 40-Hz ASSR between ASD and TD groups. However, a growing body of EEG and MEG research has applied graph-theoretical approaches to examine functional network organization in ASD more broadly (47–54). Yet, the findings have been inconsistent across these studies too, likely reflecting substantial methodological heterogeneity, including differences in recording conditions (e.g., eyes closed, eyes open with fixation, or eyes open with visual stimulation), connectivity metrics (e.g., coherence vs. phase lag index (PLI)), graph construction methods (binary vs. weighted networks), and participant characteristics such as age and medication status. Additionally, findings have varied depending on the graph metric examined. Small-worldness (SW) is the most frequently reported metric, but its direction is not consistent across studies, with some reporting reduced SW in ASD (47, 48, 51) and others reporting enhanced SW in specific frequency bands (49, 52). Findings for CC and PL are fewer and likewise mixed (47–49, 51, 52), suggesting that graph-theoretical abnormalities in ASD may not be uniform across all aspects of network topology. Nonetheless, a pattern has emerged indicating that functional brain network differences between ASD and TD groups are more robust under conditions of sensory input than under minimal or resting conditions (47, 50, 54).

When analyses are restricted to pediatric MEG studies employing PLI-based, binary, undirected graphs constructed from source-level activity in unmedicated children—an approach consistent with the present study—only three prior investigations meet these criteria (47, 50, 54). Soma et al. (47) examined young children (60–89 months) under minimal visual input (fixation cross) and reported reduced SW in the beta band in the ASD group, with beta-band SW negatively correlated with autistic trait severity as measured by the Social Responsiveness Scale (SRS). By contrast, using visually rich stimulation (video viewing) in a comparable age range, Shiota et al. (50) reported reduced SW across multiple frequency bands (delta, theta, beta, and gamma) in children with higher autistic traits, indexed by SRS-based symptom severity. This contrast suggests that sensory input amplifies group-level differences in functional network topology, revealing abnormalities that may be less apparent under minimal stimulation. This interpretation was supported by Hirosawa et al. (54), who demonstrated that visual stimulation induced systematic changes in graph measures in both children with ASD and TD children. Collectively, these pediatric graph-theoretical findings suggest that while ASD–TD differences may be subtle or frequency-specific under minimal input conditions, robust group differences in functional brain network organization emerge under active sensory stimulation.

In summary, these findings provide a strong rationale for examining functional brain networks during the 40-Hz ASSR in children with ASD and TD children. As a paradigm that imposes a well-defined rhythmic sensory drive and robustly engages NMDA receptor–dependent inhibitory microcircuits (13, 17–23), the 40-Hz ASSR constitutes a theoretically grounded condition in which group differences in functional network organization between children with ASD and TD children are expected to be reliably detectable. Therefore, in this study, we examined source-level functional brain networks during the 40-Hz ASSR using MEG and graph-theoretical analysis in 19 children with ASD and 34 TD children aged 5–8 years. Based on prior graph-theoretical studies of functional brain networks in ASD, we hypothesized that children with ASD would exhibit reduced SW, lower CC, and longer characteristic PL during the 40-Hz ASSR. Specifically, we tested the following hypotheses (1): compared with TD children, children with ASD show lower SW, lower CC, and longer PL in functional brain networks during the 40-Hz ASSR; and (2) lower SW, lower CC, and longer PL are associated with greater ASD trait severity.

## 2 Materials and methods

### 2.1 Design and participants

In this prospective observational study, we recruited children aged 5–8 years with ASD, along with their TD peers, to obtain MEG recordings. The ASD group comprised 33 children recruited from Kanazawa University and its affiliated hospitals. ASD diagnoses were made according to the Diagnostic and Statistical Manual of Mental Disorders, Fourth Edition (DSM-IV) criteria (1) and confirmed by experienced psychiatrists and psychologists using the Diagnostic Interview for Social and Communication Disorders (DISCO) (55) or the Autism Diagnostic Observation Schedule, Second Edition (ADOS-2) (56, 57).

TD children were recruited through flyers and an institutional website. Inclusion criteria were the absence of known developmental, psychiatric, or neurological diagnoses based on parent reports. The control group included 52 TD children with no known behavioral or language difficulties. Family history of ASD was not screened for in the control group.

Further, children were excluded if they met any of the following criteria (1): sensory impairments (blindness or deafness) (2), intellectual disabilities (see Section 2.2 for details), or (3) incomplete MEG or magnetic resonance imaging (MRI) data. All participants were of Japanese ethnicity.

This research was conducted as part of the Bambi Plan at the Kanazawa University Research Center for Child Mental Development, a long-term developmental neuroscience project aimed at elucidating early neurophysiological and behavioral profiles associated with autism and related developmental conditions by ongoing recruitment and assessment of children and families (https://kodomokokoro.w3.kanazawa-u.ac.jp/en/). Given this recruitment approach, some of the participants in the present study overlapped with those in our previous investigation (34).

The prior study focused on sensor-level characterization of 40-Hz ASSRs, specifically examining event-related spectral perturbation (ERSP; event-related percentage changes in signal magnitude) and inter-trial phase coherence (ITPC; a measure of trial-to-trial phase consistency) within individual brain regions. By contrast, although building on this previous work, the present study addressed a distinct research question by examining source-level functional brain networks induced during 40-Hz auditory stimulation. Accordingly, none of the results reported in this paper overlap with those presented in the earlier work, and the analytical focus and objectives of the two studies are clearly differentiated.

### 2.2 Assessment of intelligence and severity of autism symptoms

To evaluate intellectual functioning, we administered the Kaufman Assessment Battery for Children (K-ABC) or its second edition (K-ABC-II) to all participants depending on the time of assessment and test availability (58, 59). The K-ABC provides a Mental Processing Scale (MPS) that assesses problem-solving skills through simultaneous and sequential processing tasks, whereas the K-ABC-II offers the Mental Processing Index (MPI), which is conceptually similar to the original K-ABC MPS, assessing general mental processing abilities. As our study focused on children with ASD but without intellectual disabilities, we set an inclusion criterion of a score of ≥70 on these scales. This threshold aligns with the standard diagnostic criteria, distinguishing intellectual disability from average intellectual functioning (1).

To assess the severity of autism symptoms, parents completed the SRS or its second edition (SRS-2) (60, 61). The SRS and SRS-2 provide a continuous measure of social ability, ranging from impaired to above average, rather than a categorical ASD diagnosis. Higher scores are associated with greater social impairment. Because the SRS measures autism traits along a spectrum, it can identify both milder ASD symptoms as well as social impairments in individuals without ASD.

### 2.3 ASSR

The procedure for the ASSR session was identical to that employed in our previous study (34).

Participants received auditory stimulation designed to elicit an ASSR. Auditory stimuli were delivered binaurally via stereo earphones (ER-30; Etymotic Research Inc., Chicago, IL, USA) connected to a pair of long silicone tubes. The distal ends of the tubes were secured with tape to the upper left and right sides of the MEG dewar. From our prior experience, we learned that most children are uncomfortable wearing in-ear earbuds; thus, earbuds were not used in this study.

The stimuli consisted of click trains composed of brief 1-kHz sine-wave clicks presented at either 20 or 40 Hz. Each stimulus train was presented binaurally at 70 dB SPL for 1,000 ms, with an inter-trial interval of 1,000 ms that included a random jitter of −100 to +100 ms. Stimulus presentation was controlled using Presentation software (version 13.1; Neurobehavioral Systems, CA, USA) running on Windows XP. The total duration of the MEG ASSR session was approximately 7 min.

### 2.4 MEG data acquisition

MEG data were recorded using a 151-channel Superconducting Quantum Interference Device (SQUID) whole-head coaxial gradiometer system (PQ 1151R; Yokogawa/KIT, Kanazawa, Japan) housed in a magnetically shielded room (Daido Steel Co., Ltd., Nagoya, Japan). To optimize sensor positioning for children’s heads and minimize movement, we used a custom-made child-sized MEG system (62). MEG signals were low-pass filtered at 500 Hz and sampled at 2,000 Hz. Recordings were conducted under an eyes-open condition—participants watched a silent video projected onto a screen while lying supine on a bed.

To enhance engagement and minimize movement, each child selected a preferred video from a set of popular programs. Given that children with ASD may experience heightened sensory sensitivity, this approach helped reduce anxiety by providing a sense of control, minimized movement artifacts, improved data quality, and increased compliance, ultimately leading to higher-quality MEG recordings. Although this strategy may have compromised the consistency in visual stimuli across participants to some extent, the benefits of reducing stress and obtaining cleaner recordings outweighed this limitation. A staff member remained in the room to encourage stillness. Recordings were conducted between 11:00 AM and 3:00 PM. No participant showed clear signs of drowsiness based on MEG waveforms.

### 2.5 MRI

Structural brain images were acquired using a 1.5 Tesla (T) MRI scanner (SIGNA Explorer; GE Healthcare, USA) with a T1-weighted gradient-echo sequence incorporating the Silenz pulse sequence. This specialized sequence minimizes acoustic noise and shortens scan times, making it particularly suitable for pediatric populations (12, 13). The imaging parameters were as follows: repetition time (TR) = 435.68 ms, echo time (TE) = 0.024 ms, flip angle = 7°, field of view = 220 mm, matrix size = 256 × 256 pixels, and slice thickness = 1.7 mm, yielding a total of 130 transaxial images. Although this protocol resulted in slightly lower spatial resolution due to the thicker slices and lower matrix size, it provided sufficient anatomical reference while minimizing scan duration to enhance participant compliance.

### 2.6 Co-registration of MEG and MRI images

Co-registration of MEG and MRI images was performed using specific anatomical markers. Four key reference points were identified in both modalities: the midline frontal point, vertex, and bilateral mastoid processes. For MEG, magnetic field-generating coils served as markers, while for MRI, lipid capsules were used due to their high-intensity appearance in the images. Additionally, anatomical landmarks such as the mastoid processes, nasion, and skull surface were manually identified on MRI scans. To enhance accuracy, 20 additional points were marked for each participant, ensuring precise alignment between MEG and MRI data.

### 2.7 MEG data preprocessing

MEG data were processed using Brainstorm (63), an open-source software platform distributed under the GNU General Public License. Preprocessing followed the guidelines of the Organization for Human Brain Mapping (64). The preprocessing pipeline was identical to that described by Hirosawa et al. (65) and in our previous studies (47, 54, 66).

Preprocessing involved the following steps. Noisy sensors were identified and excluded. Notch filters were applied at 60, 120, and 180 Hz to eliminate power-line interference and its harmonics. A band-pass filter of 0.5–200 Hz was then applied to retain relevant frequency components. Independent component analysis (ICA) was performed to remove ocular and cardiac artifacts, specifically targeting blink- and heartbeat-related components. Finally, data segments containing apparent motion artifacts or radio-frequency interference were visually identified and removed by one of the authors (Tetsu Hirosawa), who was blinded to participant identities.

### 2.8 Source reconstruction and segmentation

MEG data were epoched from −500 to 1,000 ms relative to auditory stimulus onset (0 ms). A head model was constructed using an overlapping-sphere algorithm with a lower-resolution cortical surface comprising approximately 15,000 vertices. To estimate sensor-level noise characteristics, baseline segments from −500 to 0.5 ms were extracted from each epoch. Prior to noise estimation, direct current (DC) offsets were removed block-wise by subtracting the mean value of each channel within each recording block. Baseline segments from individual trials were then concatenated, and a sample noise covariance matrix was computed for each session.

Source reconstruction was performed using a distributed minimum-norm solution with standardized low-resolution brain electromagnetic tomography (sLORETA) normalization, incorporating the session-specific noise covariance matrix. We selected this approach *a priori* because the present study aimed to estimate distributed cortical activity across atlas-defined regions for subsequent regional aggregation and graph-theoretical analysis, rather than to localize a small number of focal generators. In this context, sLORETA provides a distributed source estimate with reduced depth bias and favorable spatial localization properties, while remaining reasonably robust to sensor noise (67).

Estimated source time series were grouped into 68 cortical regions of interest according to the Desikan–Killiany atlas, using principal component analysis to derive a representative signal for each region. Following source estimation and regional aggregation, baseline correction was applied at the source level by subtracting the mean value of the pre-stimulus interval (−500 to 0.5 ms) from each regional time series across the entire epoch.

### 2.9 Time-frequency analysis

For each participant, epoched MEG signals were first averaged across all trials to obtain an event-related potential waveform. Time–frequency decomposition was subsequently applied to this averaged waveform. Specifically, time–frequency representations were computed using Morlet wavelets with a central frequency of 2 Hz, with the mother wavelet defined to yield a temporal resolution of 3 s at the central frequency. ERSP values were calculated over a frequency range of 10–50 Hz.

For each frequency, spectral power was baseline-normalized relative to the pre-stimulus interval (−200 to 0 ms). Baseline correction was performed on a frequency-by-frequency basis by computing the mean power during the baseline period and expressing post-stimulus power changes as the percentage deviation from this baseline, according to the following formula:


$$\text{ERSP}(t,f)=\frac{P(t,f)-\langle P_{\text{baseline}}(f)\rangle }{\langle P_{\text{baseline}}(f)\rangle }\times 100$$

Here, P(t,f)denotes the instantaneous spectral power at time t and frequency f, obtained by computing the Morlet wavelet transform and taking the squared magnitude of the complex coefficient. $\langle \;P_{\text{baseline}}(f)\rangle$ denotes the baseline spectral power at frequency f, obtained by averaging P(t,f) over the pre-stimulus interval from −200 to 0 ms.

This normalization highlights stimulus-related spectral modulations while accounting for inter-frequency differences in baseline power. ERSP maps were computed at the individual-subject level and subsequently averaged across participants to obtain grand-average ERSP representations. All time–frequency analyses were performed using the default Morlet wavelet implementation in Brainstorm.

In addition to spectral power, ITPC was computed to quantify the consistency of oscillatory phase alignment across trials at each time–frequency point. ITPC reflects the degree to which the phase of neural oscillations is time-locked to stimulus onset, independent of power changes.

For each trial, the Morlet wavelet transform was applied to obtain the complex time–frequency coefficient $X_{k}(t,f)$ at time t and frequency f, where k indexes the trials. The instantaneous phase was extracted by normalizing each complex coefficient to unit magnitude, as follows:


$$\frac{X_{k}(t,f)}{\left|X_{k}(t,f)\right|}$$

Subsequently, ITPC was computed as the magnitude of the average phase vector across trials, as follows:


$$\text{ITPC}(t,f)=|\frac{1}{N}\sum_{k=1}^{N}\;\frac{X_{k}(t,f)}{\left|X_{k}(t,f)\right|}|$$

where N denotes the number of trials. ITPC values range from 0 to 1, with higher values indicating greater phase consistency across trials at a given time–frequency point. Unlike ERSP, ITPC does not require baseline normalization, as it is inherently normalized with respect to amplitude. ITPC maps were computed at the individual-subject level and subsequently averaged across participants to obtain grand-average ITPC representations. All ITPC analyses were performed using the default Morlet wavelet implementation in Brainstorm, using the same time–frequency parameters as those applied for ERSP computation.

As a supplementary analysis of conventional ASSR measures, ERSP and ITPC in the bilateral transverse temporal gyri were also compared between the ASD and TD groups across the time and frequency parameters. Further methodological details are presented in the **Supplementary Materials**.

### 2.10 PLI as a connectivity measure

Reconstructed source signals may exhibit spurious interactions due to volume conduction or field spread, which can produce artificial synchrony, particularly at zero-lag phase differences (68, 69). To mitigate such zero-lag effects and focus on physiologically meaningful connectivity, we employed the PLI—a mixing-insensitive interaction metric that reduces the influence of volume conduction by emphasizing consistent nonzero phase lags between signals (70). Additionally, PLI was selected *a priori* to maintain methodological consistency with our prior pediatric MEG studies in ASD using comparable source-level graph-theoretical pipelines (47, 50, 54).

In our previous study using the same stimulation protocol and MEG recording conditions (34), a prominent ASSR peak was observed around 40 Hz, extending into adjacent frequencies. Accordingly, in the present study, we selected a frequency range of 32–48 Hz, with an additional 2-Hz margin on each side (i.e., 30–50 Hz in total), to capture the full extent of the 40-Hz ASSR-related brain network. This frequency range is identical to that used in our previous study employing 40-Hz ASSR and graph-theoretical measures (65), ensuring comparability across studies.

For each epoch, source-level signals were band-pass filtered within the selected frequency range, and the instantaneous phase of each signal was computed using the Hilbert transform. Phase differences, $\Delta \phi (t_{k})$, were then calculated for each pair of sources at each time point *t_k_* (where k = 1, 2, …, N, and N denotes the number of time points per epoch). The PLI between two signal sources within an epoch was defined as follows (70):


$$PLI=|\frac{1}{N}\sum_{k=1}^{N}sign[\text{Δ}\phi (t_{k})]|$$

Here, the sign function is defined as +1 when $\Delta \phi (t_{k})$ > 0, −1 when $\Delta \phi (t_{k})$ < 0, and 0 when $\Delta \phi (t_{k})$ = 0. PLI values range from 0 to 1; values closer to 1 indicate a consistent nonzero phase lag between two signals over time, reflecting robust phase synchronization, and values near 0 indicate weak or no consistent phase relationship. Notably, PLI does not provide information about the directionality of phase lead or lag, but rather quantifies the consistency of phase asymmetry. PLI was computed for all pairs of source signals within the selected frequency band to estimate functional connectivity.

### 2.11 Graph construction and metrics

To characterize the topological properties of the 40-Hz ASSR-related functional brain network, we employed graph-theoretical analysis, representing the network as a graph composed of nodes and edges. The network comprised 68 nodes corresponding to cortical regions defined by the Desikan–Killiany atlas, with weighted edges derived from pairwise PLI values.

For each epoch, an undirected weighted functional connectivity matrix of size 68 × 68 was constructed at the source level. These matrices were then averaged across all epochs for each participant. To reduce spurious connections and simplify network topology, the averaged connectivity matrices were binarized using a proportional threshold of κ = 0.20, retaining the strongest 20% of connections. We selected this threshold to maintain consistency with prior MEG studies in pediatric ASD populations using comparable source-level graph-theoretical approaches (47, 50, 54) and because binary proportional thresholding controls for differences in graph density, which can strongly influence graph-theoretical measures. This approach also allowed us to focus on the topological organization of the strongest connections while reducing the influence of weak connections that may be less stable.

For sensitivity analysis, we repeated the graph construction procedure and primary regression analyses for CC, PL, and SW across a range of proportional thresholds surrounding the main analysis threshold (κ = 0.12, 0.14, 0.16, 0.18, 0.22, 0.24, 0.26, 0.28, and 0.30). These threshold-sensitivity analyses were interpreted descriptively. The secondary regression model relating PL to SRS total T-scores was also repeated across the same thresholds for exploratory analysis.

For the resulting binary graphs, we computed commonly used graph metrics, including the CC, characteristic PL, and SW, using the Brain Connectivity Toolbox (BCT; version 2019-03-03; https://sites.google.com/site/bctnet/) (71). Mathematical definitions of these metrics are provided in previous literature (71, 72). The CC quantifies the tendency of nodes to form local clusters and reflects the degree of segregated neural processing within the network (72).

The characteristic PL is defined as the average shortest-path distance between all pairs of nodes and serves as an index of global information integration efficiency. After binarizing the adjacency matrices, we computed the shortest-path distance matrix D using the distance_bin function of BCT, where each element *D_ij_* represents the length of the shortest path between nodes i and j and is set to ∞ when no connecting path exists. We then applied the charpath function of BCT with parameters charpath(D, 0, 0), where the option infinite_dist = 0 instructs the function to exclude all ∞ distances and compute the average over finite distances only. Accordingly, the characteristic PL was calculated as follows:


$$\text{PL}=\frac{1}{\left|\{(i,j)D_{ij}<\infty ,i<j\}\right|}\sum_{D_{ij}<\infty ,i<j}D_{ij}$$

This approach effectively approximated computation within the largest connected component, as the thresholded binary graphs typically contained one dominant connected component encompassing most nodes, with only a small number of isolated nodes or minor subcomponents. Importantly, we did not explicitly remove isolated components; rather, we relied on charpath(D,0,0) to exclude ∞ distances while retaining all finite-distance node pairs. This procedure aligns with prior MEG-based ASD studies (47, 49, 50, 54) and with Brainstorm’s implementation of BCT measures, thereby ensuring methodological consistency and comparability.

SW captures the balance between local specialization and global integration, a hallmark of efficient brain networks (72). SW was computed as the ratio of the normalized CC to the normalized characteristic PL relative to equivalent random networks (73). For each graph, we generated 1,000 random networks by rewiring edges 10 times while preserving the number of nodes and edges. The mean CC (CC_rand_) and characteristic PL (PL_rand_) were calculated across these random networks. Normalized metrics were defined as follows:


$${\text{CC}}_{\text{norm}}=\frac{\text{CC}}{{\text{CC}}_{\text{rand}}}\text{ , }P{\text{L}}_{\text{norm}}=\frac{\text{PL}}{{\text{PL}}_{\text{rand}}}$$

and SW was calculated as follows:


$$\text{SW}=\frac{{\text{CC}}_{\text{norm}}}{{\text{PL}}_{\text{norm}}}$$

### 2.12 Statistical analyses

Statistical analyses were performed using Stata (version 17.0; StataCorp LLC, College Station, TX, USA). Group differences in continuous participant characteristics, including age in months, SRS scores, epoch counts, and mental processing abilities as measured by the K-ABC, were assessed using two-tailed Wilcoxon rank-sum tests. Sex differences were examined using the chi-square test.

Our primary objective was to examine the effects of diagnosis (ASD vs. TD) on three graph measures derived from the 40-Hz ASSR functional brain network: SW, CC, and characteristic PL. To assess diagnostic effects, we fitted separate linear regression models for each graph measure (SW, CC, and PL). Each model included diagnosis (ASD vs. TD), age in months, sex, and mental processing abilities, as measured by the K-ABC, as covariates. The primary regression models were specified as follows:


$$Y_{i}={\text{β}}_{0}+{\text{β}}_{1}{\text{Diagnosis}}_{\text{i}}+{\text{β}}_{2}{\text{Age}}_{\text{i}}+{\text{β}}_{3}{\text{Sex}}_{\text{i}}+{\text{β}}_{4}{\text{MentalProcessingScale}}_{\text{i}}+{\text{ϵ}}_{i}$$

where $Y_{i}$ denotes each graph-theoretical outcome (SW, CC, or PL) for participant i. Bonferroni correction was applied to the primary diagnostic-effect analyses to account for multiple testing across the three pre-specified graph measures, and statistical significance for these primary analyses was set at p < 0.0167 (0.05/3).

If a significant effect of diagnosis was observed for any graph measure, we subsequently examined whether that graph measure was associated with autistic traits, indexed by the SRS total T-score. For this secondary dimensional analysis, we fitted a linear regression model predicting SRS total T-scores from the graph measure of interest, diagnosis, and their interaction term, while adjusting for age in months, sex, and mental processing abilities, as measured by the K-ABC. The secondary regression model was specified as follows:


$${\text{SRS}}_{\text{i}}={\text{β}}_{0}+{\text{β}}_{1}{\text{GraphMeasure}}_{\text{i}}+{\text{β}}_{2}{\text{Diagnosis}}_{\text{i}}+{\text{β}}_{3}({\text{GraphMeasure}}_{\text{i}}\times {\text{Diagnosis}}_{\text{i}}+{\text{β}}_{4}{\text{Age}}_{\text{i}}+{\text{β}}_{5}{\text{Sex}}_{\text{i}}+{\text{β}}_{6}\text{MentalProcessingScale}+{\text{ϵ}}_{i}$$

where ${\text{GraphMeasure}}_{\text{i}}$ denotes the graph-theoretical metric of interest for participant i. This model was used to evaluate both the overall association between the graph measure and autistic traits and whether that association differed by diagnosis. For the secondary analysis, statistical significance was evaluated at p < 0.05, and the results were interpreted as secondary findings.

To examine whether the association between cognitive ability and the outcomes differed by diagnosis, we conducted additional sensitivity analyses by including a diagnosis × mental processing ability interaction term in the primary regression models for CC, SW, and PL and in the secondary regression model predicting SRS total T-scores.

To evaluate model assumptions for regression models central to our primary inferences, we conducted a limited set of diagnostic checks. Residual distributions were visually inspected using histograms to assess approximate normality and the absence of pronounced skewness or kurtosis. Homoscedasticity was evaluated by plotting residuals against fitted values. While residual distributions appeared approximately normal, evidence of heteroscedasticity was observed in some models. Therefore, heteroscedasticity-robust standard errors were used throughout (74).

Quantitative head-motion estimates were not available; hence, they were not included as covariates in the regression models.

## 3 Results

### 3.1 Participants

Of the 33 children with ASD initially recruited, data from 14 were excluded from the analyses: two boys were unable to complete the psychological assessment; five boys met the criteria for intellectual disability, as indicated by a K-ABC MPS score below 70; and five boys and two girls exhibited excessive noise in their MEG recordings or were unable to complete MRI acquisition.

Of the 52 children initially enrolled in the TD group, data from 18 were excluded from the analyses: one boy and two girls were unable to complete the psychological assessment, and eight boys and seven girls exhibited excessive noise in their MEG recordings or were unable to complete MRI acquisition.

Consequently, the final sample comprised 19 children with ASD (14 boys and 5 girls) and 34 TD children (26 boys and 8 girls).

The age range of the ASD group was 60–96 months, whereas that of the TD group was 60–91 months. There were no significant group differences in sex distribution, age, or number of available epochs. By contrast, cognitive ability, assessed using the K-ABC MPS (or the MPI for participants assessed with the K-ABC-II), was significantly lower in the ASD group than in the TD group (z = 3.15, p = 0.002). Total SRS scores were significantly higher in the ASD group than in the TD group (z = −4.56, p < 0.001). These demographic and behavioral characteristics are summarized in **Table 1**.

**Table 1:** Demographic and behavioral characteristics of typically developing (TD) and autism spectrum disorder (ASD) groups.

Characteristic	TD	ASD	χ^2^ or *z*	*P*
N	34	19		
Sex (% male)†	26	14	0.05	0.82
Age‡	68 (62–75)	71 (66–80)	-1.47	0.14
Epoch number‡	206.5 (175–226)	182 (91–221)	1.19	0.24
K-ABC MPS/K-ABC-II MPI‡	113 (97–124)	87 (78–98)	3.15	0.002
SRS total t-score‡	45 (43–50)	67 (57–77)	-4.56	<0.001

Continuous variables are presented as median (interquartile range [IQR]), and categorical variables are presented as counts.

† Sex distribution was compared using the χ² test.

‡ Continuous variables were compared using the Wilcoxon rank-sum test.

ASD, autism spectrum disorder; TD, typically developing; SRS, Social Responsiveness Scale; K-ABC, Kaufman Assessment Battery for Children; MPS, Mental Processing Scale; MPI, Mental Processing Index; χ², chi-square statistic.

### 3.2 Validation of the 40-Hz ASSR paradigm

**Figure 1:**
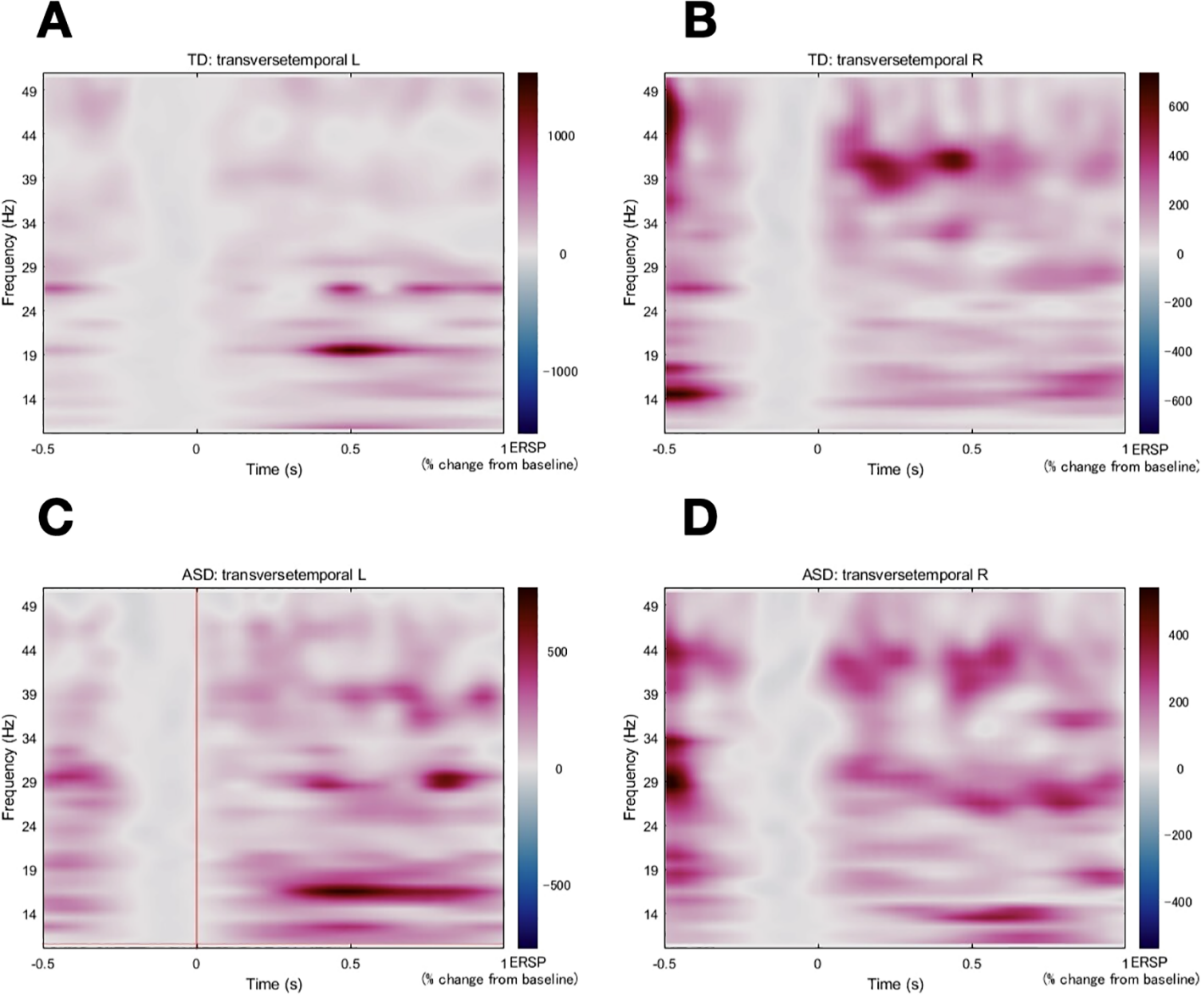
Event-related spectral perturbation (ERSP) during 40-Hz auditory stimulation. Grand-averaged time–frequency representations of ERSP elicited by the 40-Hz auditory stimulus are shown for typically developing (TD) children [**(A)** left transverse temporal gyrus; **(B)** right transverse temporal gyrus] and children with ASD [**(C)** left; **(D)** right]. Stimulus-related ERSP changes were observable in both groups, although their frequency distribution and visual prominence varied across panels. In particular, 40-Hz-related activity was more evident in panels **(B, D)** than in panels **(A, C)**. In all panels, stimulus onset corresponds to 0 ms.

**Figure 2:**
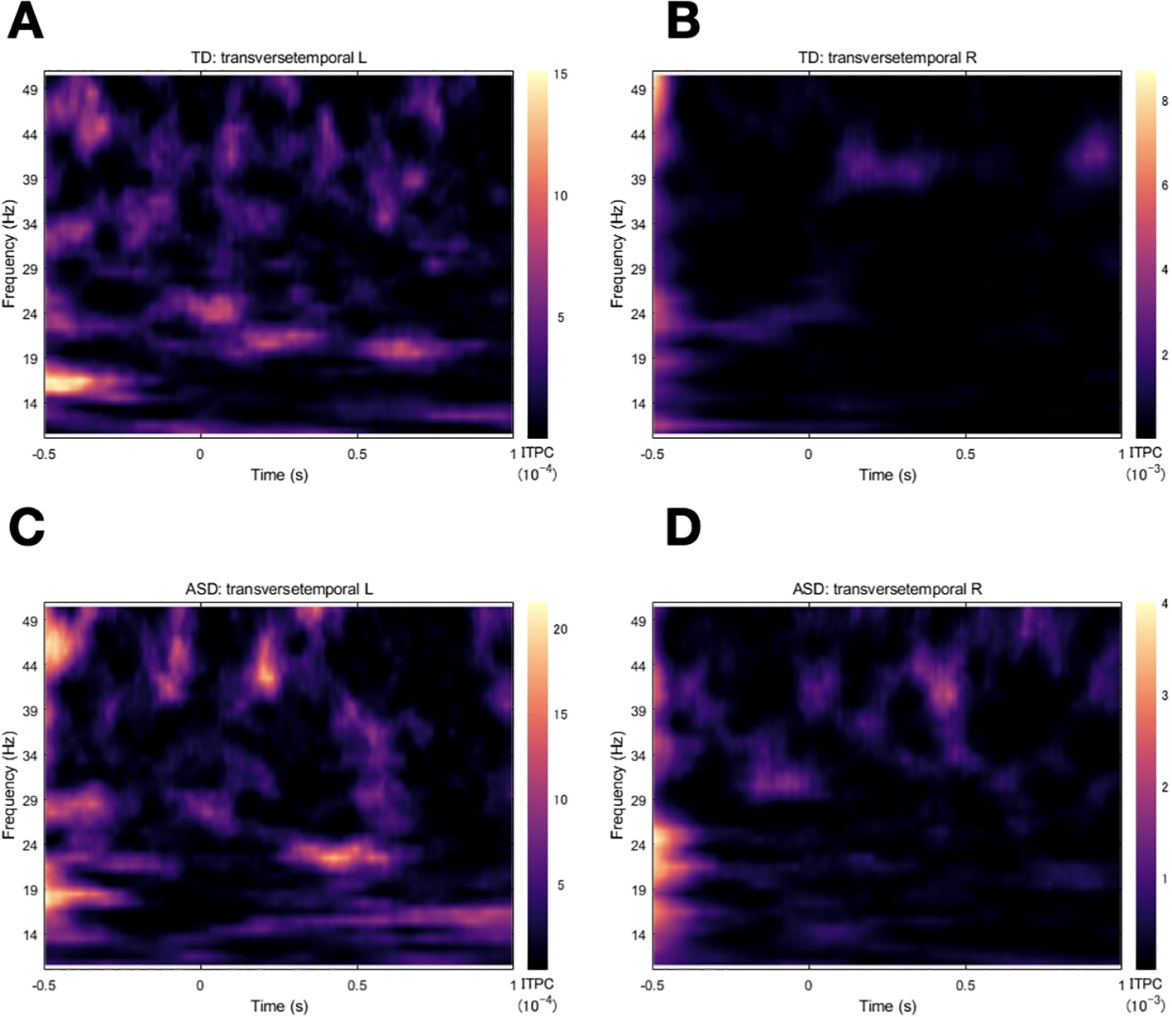
Inter-trial phase coherence (ITPC) during 40-Hz auditory stimulation. Grand-averaged time–frequency representations of ITPC elicited by the 40-Hz auditory stimulus are shown for TD children [**(A)** left transverse temporal gyrus; **(B)** right transverse temporal gyrus) and children with ASD [**(C)** left; **(D)** right]. Time–frequency changes in ITPC were observable in both groups, but the visual prominence of 40-Hz-related activity varied across panels and was not uniformly distinct from surrounding activity. In some panels, lower-frequency activity, including subharmonic components around 20 Hz, appeared visually prominent. In all panels, stimulus onset corresponds to 0 ms.

**Figures 1** and **2** show the grand-averaged time–frequency representations of ERSP and ITPC in response to the 40-Hz stimulus. In the bilateral transverse temporal gyri, stimulus-related ERSP changes are observable in both TD children (**Figures 1A, B**) and children with ASD (**Figures 1C, D**), although their frequency distribution and visual prominence vary across panels. In particular, 40-Hz-related activity is more evident in **Figures 1B, D** than in **Figures 1A, C**. In addition to spectral power, ITPC was computed to quantify the consistency of oscillatory phase alignment across trials at each time–frequency point. In both TD children (**Figures 2A, B**) and children with ASD (**Figures 2C, D**), time–frequency changes in ITPC are observable, but the visual prominence of 40-Hz-related activity varies across panels and is not uniformly distinct from surrounding activity. In some panels, lower-frequency activity, including subharmonic components around 20 Hz, appears visually prominent. Taken together, these figures should be interpreted as descriptive support for successful elicitation of an ASSR, rather than as strong visual evidence of a uniform 40-Hz response across all panels.

To further examine whether ASSR-related activity extended beyond the auditory cortex, we performed a descriptive inspection of source-level ERSP and ITPC across cortical regions in the full sample. This inspection indicated that 40-Hz ASSR-related activity was not limited to the bilateral transverse temporal gyri, but was also observable in several extra-auditory regions, including the ventral temporal, orbitofrontal, and occipital areas. Particularly prominent responses were observed in regions such as the entorhinal cortex, fusiform gyrus, inferior temporal cortex, orbitofrontal cortex, pericalcarine cortex, and temporal pole. These observations support the rationale for examining whole-brain functional network organization during 40-Hz auditory stimulation and are consistent with the present paradigm having successfully elicited distributed ASSR-related cortical activity. A summary of regional ERSP and ITPC findings is provided in **Supplementary Table 1**, and representative time–frequency plots are shown in **Supplementary Figures 1**-**S8**.

To supplement this descriptive inspection, we compared conventional ASSR measures between the ASD and TD groups in the bilateral transverse temporal gyri across the time and frequency parameters. Further details regarding the methodology and results for this supplementary analysis are provided in the **Supplementary Materials**. Briefly, no false discovery rate (FDR) –corrected group differences were observed for ERSP. For ITPC, only a small number of isolated time–frequency bins in the left transverse temporal gyrus survived FDR correction, without forming a coherent sustained 40-Hz pattern. Accordingly, these findings were not interpreted as evidence of a robust group difference in conventional ASSR measures and were broadly consistent with our previous report using the same paradigm in a similar age range (34).

### 3.3 Effects of diagnosis on graph-theoretical measures of the 40-Hz ASSR functional brain network

Separate multiple linear regression analyses were conducted for each graph-theoretical measure—CC, PL, and SW. Each model included diagnosis (ASD vs. TD) as the primary independent variable, with age, sex, and mental processing abilities, as measured by the K-ABC, entered as covariates. Robust standard errors were used in all analyses.

**Figure 3:**
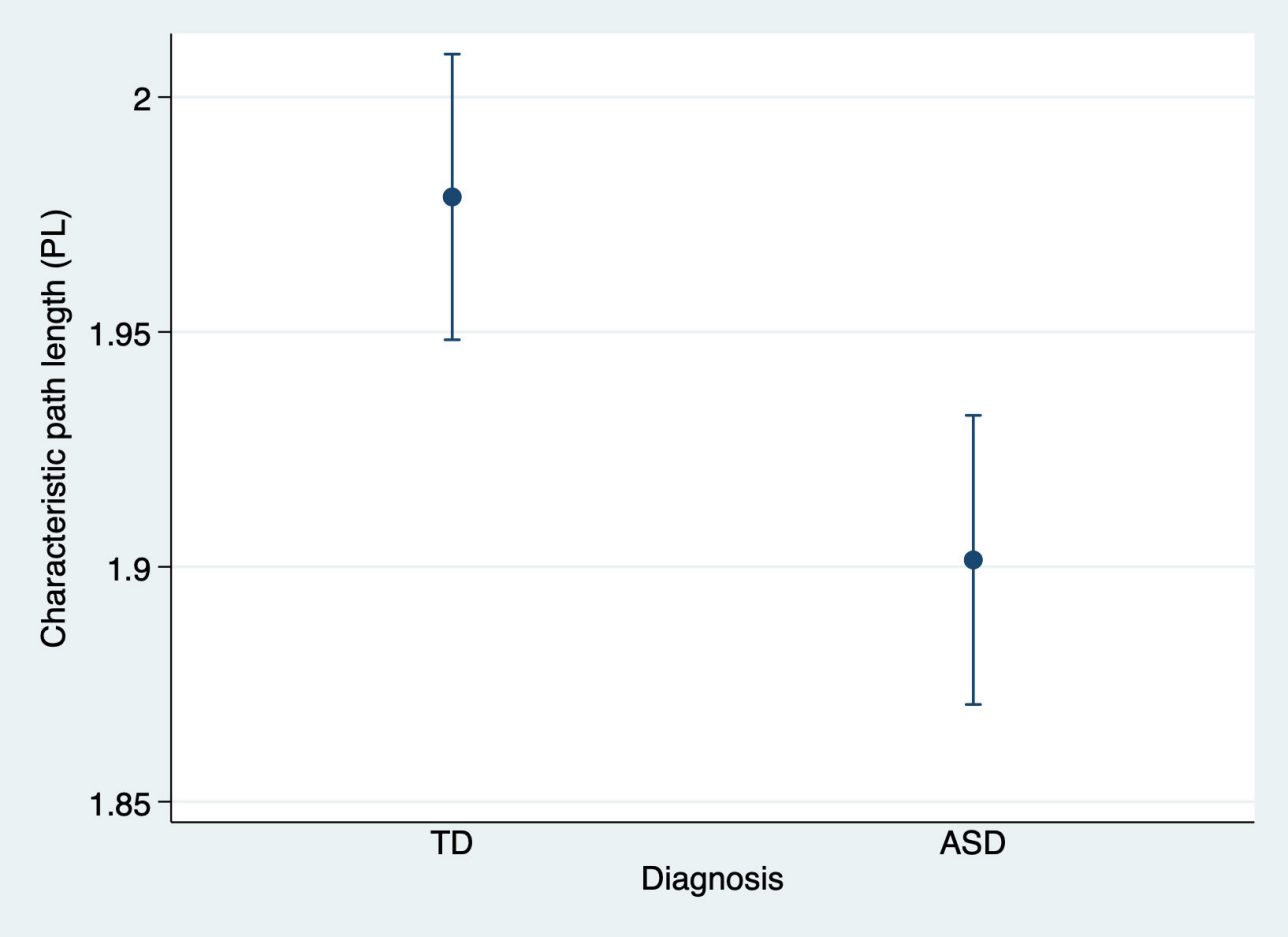
Covariate-adjusted characteristic path length (PL) by diagnosis. Predicted margins of characteristic PL derived from a multiple linear regression model including diagnosis (ASD vs. TD), age in months, sex, and mental processing abilities (K-ABC) as predictors. Points represent model-adjusted predicted values for each diagnostic group, averaged over the observed distributions of the covariates, and error bars indicate 95% confidence intervals. The ASD group showed significantly shorter characteristic PL than the TD group.

At the main analysis threshold (κ = 0.20), a significant main effect of diagnosis was observed for PL (t = −3.22, p = 0.0023), indicating shorter PL values in the ASD group compared with the TD group (**Figure 3**). By contrast, no significant effects of diagnosis were observed for CC or SW. Detailed results of these regression analyses are presented in **Table 2**.

**Table 2:** Multiple linear regression analyses of 40 Hz ASSR-related network metrics.

Variable	Coefficient	Robust SE	*t*	*p*	Significance	95% CI lower	95% CI upper	*F*	Model p value	*R^2^*
Clustering coefficient										
Diagnosis (ASD vs. TD)	-0.018	0.015	-1.220	0.228		-0.048	0.012	2.62	0.05	0.16
Age	-0.000	0.001	-0.180	0.857		-0.001	0.001			
Sex	-0.031	0.017	-1.890	0.065		-0.064	0.002			
Mental processing scale	0.000	0.000	0.690	0.495		0.000	0.001			
Characteristic PL										
Diagnosis (ASD vs. TD)	-0.077	0.024	-3.220	0.002	*	-0.125	-0.029	2.87	0.03	0.24
Age	0.000	0.001	0.270	0.792		-0.002	0.003			
Sex	0.038	0.021	1.810	0.077		-0.004	0.081			
Mental processing scale	-0.001	0.001	-2.000	0.051		-0.002	0.000			
Small-worldness										
Diagnosis (ASD vs. TD)	0.024	0.042	0.580	0.568		-0.060	0.108	0.91	0.47	0.05
Age	0.001	0.002	0.630	0.532		-0.003	0.006			
Sex	0.041	0.041	1.000	0.323		-0.042	0.124			
Mental processing scale	0.001	0.001	1.230	0.226		-0.001	0.003			

ASD, autism spectrum disorder; TD, typically developing; PL, path length. Robust SE, heteroscedasticity-robust standard error; CI, confidence interval; F, F statistic; R², coefficient of determination.

An asterisk (*) indicates *p* < 0.05.

As a threshold-sensitivity analysis, we repeated the graph construction procedure and all subsequent regression analyses across a range of proportional thresholds surrounding the main analysis threshold. Across these analyses, the effect of diagnosis on PL remained negative at all tested thresholds, indicating shorter PL values in the ASD group than in the TD group throughout. This pattern was most evident in the intermediate threshold range surrounding the main analysis threshold. By contrast, CC and SW did not show a comparable diagnosis-related pattern across thresholds. Detailed results are presented in **Supplementary Tables 2**-**S4**.

In an additional sensitivity analysis at the main analysis threshold (κ = 0.20), no significant interaction of diagnosis × mental processing ability was observed for CC, SW, or PL, indicating that the association between cognitive ability and graph-theoretical measures did not differ detectably by diagnosis (**Supplementary Table 6**).

### 3.4 Association between characteristic PL and autistic traits

After identifying a significant effect of diagnosis on PL, we examined whether PL was associated with autistic traits, indexed by SRS total T-scores. We fitted a linear regression model including PL, diagnosis, and their interaction term, while adjusting for age in months, sex, and mental processing abilities, as measured by the K-ABC.

The diagnosis × PL interaction was not statistically significant, indicating that the association between PL and autistic traits did not significantly differ between ASD and TD groups. However, a significant association was observed between PL and SRS total T-scores in the model, with shorter PL associated with greater autistic trait severity (t = −2.28, p = 0.027). Age in months was also significantly associated with SRS total T-scores (t = 2.13, p = 0.038). Detailed results of this analysis are presented in **Table 3**.

**Table 3:** Linear regression analysis of SRS total T-scores including characteristic path length (PL), diagnosis, and their interactionF.

Variable	Coefficient	Robust SE	*t*	*p*	Significance	95% CI lower	95% CI upper	*F*	Model p value	*R^2^*
SRS total t-score										
Characteristic PL	-33.386	14.645	-2.280	0.027	*	-62.866	-3.907	12.60	<0.001	0.52
Diagnosis (ASD vs. TD)	-82.113	109.480	-0.750	0.457		-302.485	138.260			
Diagnosis × Characteristic PL	50.419	57.231	0.880	0.383		-64.782	165.620			
Age	0.337	0.158	2.130	0.038	*	0.019	0.656			
Sex	-3.066	4.941	-0.620	0.538		-13.012	6.880			
Mental processing scale	-0.100	0.110	-0.910	0.366		-0.321	0.121			

ASD, autism spectrum disorder; TD, typically developing; SRS, Social Responsiveness Scale; PL, path length. Robust SE, heteroscedasticity-robust standard error; CI, confidence interval; F, F statistic; R², coefficient of determination.

An asterisk (*) indicates *p* < 0.05.

In an additional sensitivity analysis including a diagnosis × mental processing ability interaction term, no significant interaction was observed, indicating that the association between cognitive ability and SRS total T-scores also did not differ detectably by diagnosis (**Supplementary Table 7**).

## 4 Discussion

In this study, we examined how ASD diagnosis and autistic traits, as measured by the SRS, are associated with three key graph-theoretical measures—CC, PL, and SW—derived from functional brain networks during the 40-Hz ASSR in children with and without ASD. Specifically, we tested the following hypotheses (1): compared with TD children, children with ASD would show lower SW, lower CC, and longer PL in functional brain networks during the 40-Hz ASSR; and (2) lower SW, lower CC, and longer PL would be associated with greater ASD trait severity. Before conducting the graph-theoretical analyses, we confirmed that the present experimental paradigm elicited ASSR-related cortical activity. Contrary to our initial expectations, the results revealed several unexpected patterns. First, we observed a significant effect of diagnosis on PL, indicating shorter PL in functional brain networks during the 40-Hz ASSR in children with ASD compared with TD children. By contrast, no significant effects of diagnosis were observed for CC or SW. Second, in the secondary analysis, shorter PL was associated with higher SRS total T-scores in the regression model. Because the PL × diagnosis interaction was not statistically significant, this association is best interpreted as a relationship across the full sample rather than as a diagnosis-specific effect.

To our knowledge, this is the first study to demonstrate a group difference in a graph-theoretical measure between children with ASD and TD children during the 40-Hz ASSR. Notably, we observed significantly shorter PL in children with ASD than in TD children. This finding contrasts with our previous result (29), which showed no significant group differences in conventional 40-Hz ASSR measures such as spectral power and phase-locking indices. Similarly, prior studies by Edgar et al. (32) and Stroganova et al. (33) reported no significant differences in ASSR power or phase locking between children with ASD and TD children across broader age ranges (7–14 and 7–12 years, respectively). Rather than indicating that graph-theoretical analysis is inherently more sensitive than conventional ASSR measures, the present findings suggest that network-level analysis may provide complementary information about the organization of cortical responses during 40-Hz auditory stimulation. In this context, the present study may have captured ASD-related atypicalities in the 40-Hz ASSR at the level of large-scale network organization. At the same time, the interpretation of shorter PL requires caution. In graph theory, PL is a topological measure reflecting the configuration of connections within a network, and shorter PL is generally interpreted as indicating a pattern associated with greater global integration, independent of the overall strength of phase coupling (42). However, in the present study, PL was the only graph measure showing a significant diagnosis effect, whereas CC and SW did not differ significantly between groups. Accordingly, the present findings do not support a broad alteration across all aspects of network topology, but rather suggest a more selective difference in network configuration during externally driven 40-Hz auditory stimulation. The biological basis and functional significance of this finding remain to be clarified. One possibility is that atypical excitation–inhibition dynamics in ASD (14, 15) may influence large-scale network organization during rhythmic stimulation such as the 40-Hz ASSR in a manner that biases the network toward greater global integration, but this remains speculative and should be tested more directly in future studies. In this regard, it is noteworthy that a resting-state EEG study focused on fragile X syndrome (75) reported increased spatial spreading of phase-synchronized gamma-band activity, suggesting the possibility of more widespread high-frequency synchronization in neurodevelopmental conditions. This observation may be broadly consistent with the possibility of topological differences in the functional brain networks engaged during the 40-Hz ASSR in TD and ASD populations. However, such findings relate more directly to overall connectivity patterns than to PL in binarized graph topology; therefore, any link to the present PL finding remains speculative.

Although no previous studies have examined graph-theoretical measures specifically during the 40-Hz ASSR, two prior investigations (47, 50) employed highly comparable analytical frameworks—PLI-based, binary, undirected graphs constructed from source-level activity in unmedicated children—allowing for meaningful comparison with the present study. In these studies, no significant differences in PL between children with ASD and TD children were observed, either under minimal visual stimulation (dark room with fixation cross (47)) or under rich visual stimulation (video viewing (50)). Broadening the scope to studies examining functional brain network organization in ASD using graph-theoretical measures including PL, several investigations have involved experimental settings that partially overlap with ours, although none are identical. These studies differ in key methodological aspects, including participant age (adult samples), connectivity metrics (e.g., synchronization likelihood), recording modality (EEG or MEG), analysis space (sensor vs. source space), and graph construction (weighted vs. binary) (48, 49, 51, 52, 76). Among these studies, only two investigated frequency bands overlapping with the present 30–50 Hz range under resting-state conditions—Han et al. (30–45 Hz (76)) and Ye et al. (30–80 Hz (52)). Of these studies, only Han et al. reported a significant group difference in characteristic PL, with longer PL in ASD compared with TD participants, which is opposite in direction to the present findings. In contrast to these resting-state paradigms, the present findings obtained during auditory stimulation at 40 Hz suggest that ASSR-specific network engagement may reveal distinct alterations in functional network topology in ASD. Given that the 40-Hz ASSR robustly engages inhibitory microcircuits dependent on NMDA receptor–mediated and GABAergic signaling (13, 19–23), these results point to a condition-specific sensitivity of graph-theoretical measures to underlying neurobiological mechanisms. Together with prior evidence that graph-theoretical alterations in ASD can emerge under visual stimulation (54), the present findings underscore the critical role of experimental context and sensory modality in shaping functional brain network organization. Beyond visual input, auditory stimulation—particularly rhythmic stimulation at gamma frequencies—may delineate distinct aspects of network topology.

In the secondary analysis, shorter PL was associated with higher SRS total T-scores in the omnibus regression model. Because the PL × diagnosis interaction was not statistically significant, this relationship is better interpreted as an association with autistic-trait severity across the full sample rather than as a diagnosis-specific effect. Although no previous studies have directly examined the relationship between 40-Hz ASSR-related network topology and autistic traits, existing evidence provides some context for this finding. The 40-Hz ASSR has been linked to fine-grained temporal analysis of auditory input (77)—a process important for speech perception, particularly under challenging listening conditions. Consistent with this view, prior studies have demonstrated associations between 40-Hz ASSR responses and speech-in-noise perception in both younger and older adults (78). Given that atypical speech-in-noise perception is frequently reported in ASD (79), network organization during the 40-Hz ASSR may relate to behavioral variation relevant to autistic traits. In graph-theoretical terms, PL reflects the efficiency of global information transfer determined by network configuration (42), with shorter PL generally indicating a pattern of greater global integration. In this context, the observed association between shorter PL and higher SRS total T-scores suggests that greater autistic-trait severity may be associated with a more globally integrated pattern of network organization during the 40-Hz ASSR. At the same time, this interpretation warrants caution. The biological meaning of shorter PL in this context is uncertain, and the present data do not by themselves establish whether this pattern reflects adaptive, maladaptive, or compensatory dynamics. This caution is especially important because PL was the only graph-theoretical measure showing a diagnosis effect in the primary analysis. Nevertheless, the potential importance of PL as a modifiable 40-Hz ASSR-derived network property is supported by our previous work focused on healthy adults using weighted, undirected graphs (65), in which characteristic PL was significantly reduced following transcranial direct current stimulation (tDCS) relative to sham stimulation. Together, these findings suggest that network integration during the 40-Hz ASSR may represent a meaningful systems-level correlate of autistic traits and a possible target for future mechanistic or interventional studies.

Age in months was also significantly associated with SRS total T-scores in the omnibus model. This finding should be interpreted cautiously. Prior work has suggested that age can influence SRS raw scores in ASD (80), whereas, to our knowledge, there is no well-established literature specifically examining age effects on SRS total T-scores in this context. Accordingly, the developmental meaning of the present age effect remains uncertain.

Several methodological factors should be considered when interpreting the present findings. A substantial proportion of participants were excluded during data preprocessing, with 42% of children in the ASD group and 35% in the TD group excluded due to intellectual disability, incomplete psychological assessment, excessive motion artifacts during MEG acquisition, or missing MRI data. Although such attrition likely reflects the practical challenges inherent in acquiring high-quality neuroimaging data from young children—particularly those from clinical populations—it may nonetheless constrain the generalizability of the results. In particular, the reduced sample size, together with the imbalance between groups, may have lowered statistical power and limited sensitivity to detect smaller effects. As noted by Ioannidis (81, 82), underpowered studies are more likely to miss true effects, overestimate effect sizes when findings are significant, and yield findings with lower replicability. In our study, these concerns are particularly relevant to the main effect of diagnosis on PL, which also informed downstream analyses. Although this effect reached statistical significance, it should be considered preliminary and interpreted with caution. While we consider these exclusions to be largely procedural rather than systematic, caution is warranted when extrapolating the findings to broader populations of children with and without ASD. Another limitation is the absence of quantitative head-motion estimates. During acquisition, movement was minimized as much as possible by recording participants in a supine position, allowing each child to watch a preferred silent video to reduce anxiety and improve compliance, and having a staff member remain in the room to encourage stillness. Furthermore, visually apparent motion artifacts were removed during preprocessing, and participants with excessive noise were excluded. However, because quantitative head-motion estimates were unavailable, residual motion effects could not be modeled directly and, therefore, cannot be fully ruled out. In addition, the TD group was identified based on parent reports and the absence of known clinical diagnoses. Although none of the TD participants were reported to have language or behavioral difficulties, standardized instruments were not employed to formally screen for subclinical neurodevelopmental or psychiatric traits. Consequently, the presence of subtle characteristics that could influence functional brain network organization cannot be entirely ruled out. With respect to clinical characterization, participants with known comorbidities or current medication use were excluded; however, prior medication history was not systematically assessed, and comorbidity status was not independently verified beyond referral diagnosis in the ASD group or parent reports in the TD group. These factors may introduce residual confounding effects on the functional connectivity patterns and graph-theoretical metrics reported in this paper. Another limitation concerns the sample composition. All participants were of Japanese ethnicity, which may limit the extent to which the present findings can be generalized to populations with different racial or ethnic backgrounds. Replication in more diverse cohorts will be essential to establish the broader applicability of these results. Furthermore, participant vigilance during MEG acquisition was not objectively monitored. Although no overt signs of drowsiness were observed based on visual inspection of MEG waveforms, this assessment—conducted by a single author—was necessarily subjective and may not have detected transient or subtle fluctuations in alertness. Variations in vigilance are a well-recognized challenge in resting-state paradigms, particularly in pediatric samples (83, 84), and can influence both spectral power (85) and functional connectivity, especially when coherence-based measures are used (86). While PLI-based connectivity metrics and derived network topology are considered relatively robust to drowsiness-related confounds (87, 88), the absence of objective vigilance measures (e.g., electrooculography, eye tracking, or behavioral probes) remains a limitation. Another limitation concerns source estimation. Because MEG source reconstruction is inherently model-dependent, the present findings may vary to some extent depending on the inverse solution used. Although sLORETA was selected *a priori* for the reasons described above, future studies should examine the reproducibility of these network findings using alternative source reconstruction approaches, such as beamforming methods. In addition, the use of binary graph construction entails its own methodological trade-offs. While the binary PLI-based pipeline used in this study is consistent with prior pediatric MEG studies on ASD and facilitates comparison across participants by controlling graph density, it also discards information about connection strength. Thus, weighted graph approaches may capture complementary aspects of network organization and may be less sensitive to hard thresholding. Moreover, proportional thresholding can enforce equal density across groups and thereby alter the representation of weaker but potentially meaningful connections. Although our threshold-sensitivity analyses showed that the diagnosis-related PL effect remained directionally consistent across a range of thresholds, future work should examine whether the present findings are reproducible using weighted-graph approaches. A further methodological limitation concerns the choice of connectivity metric. Although PLI was selected *a priori* for the reasons described above, alternative phase-based measures such as weighted phase lag index (wPLI) and debiased weighted phase lag index (dwPLI) may be less sensitive to additional uncorrelated noise sources and may, in some settings, provide greater statistical power to detect changes in phase synchronization (89). Future work should therefore examine whether the present findings are reproducible across alternative connectivity metrics. Finally, we did not include a baseline functional brain network for direct comparison with the 40-Hz ASSR condition. From a practical standpoint, the relatively short inter-stimulus interval (1000 ms with a random jitter of −100 to +100 ms) made it difficult to define a stimulus-independent baseline that was not influenced by residual gamma-band activity or lingering network-level effects from the preceding stimulation. Beyond this design constraint, baseline correction at the level of functional brain networks poses conceptual challenges. Graph-theoretical measures such as the CC, characteristic PL, and SW are nonlinear metrics (41–43), and their interpretation under subtraction or normalization relative to a baseline network is not straightforward. Moreover, resting or pre-stimulus network organization is known to differ between children with ASD and TD children and may be modulated by arousal state. Thus, baseline normalization may obscure diagnostically meaningful network properties rather than isolate stimulation-specific effects. Given that the primary objective of the present study was to characterize functional network organization under rhythmic auditory stimulation at 40 Hz, we focused on absolute network properties during the ASSR condition. Future investigations incorporating longer inter-stimulus intervals or independent resting-state recordings will be necessary to further disentangle baseline-related and stimulation-specific contributions to network topology.

In conclusion, the present study suggests that functional brain network topology during the 40-Hz ASSR may provide complementary information about ASD-related neural alterations beyond conventional ASSR measures. Specifically, we identified shorter PL in children with ASD than in TD children, as well as an association between shorter PL and greater autistic-trait severity across the full sample. These findings suggest that network-level organization during externally driven gamma-band synchronization is relevant to both diagnostic status and variation in autistic-trait severity across the sample. Together, our results support the value of graph-theoretical analysis of the 40-Hz ASSR for characterizing atypical network organization in ASD. Future studies combining task-based and resting-state paradigms, longitudinal designs, and mechanistic or interventional approaches will be important for clarifying the developmental and clinical significance of these network alterations.

## Data Availability

The original contributions presented in the study are included in the article/**Supplementary Material**. Further inquiries can be directed to the corresponding author.
