# Supplementary figures and images for: Functional brain network organization during the 40-Hz auditory steady-state response in children with and without autism spectrum disorder
Source: Front Psychiatry. 2026 Jun 9;17:1804124. doi: 10.3389/fpsyt.2026.1804124 (PMC13288207; doi:10.3389/fpsyt.2026.1804124)

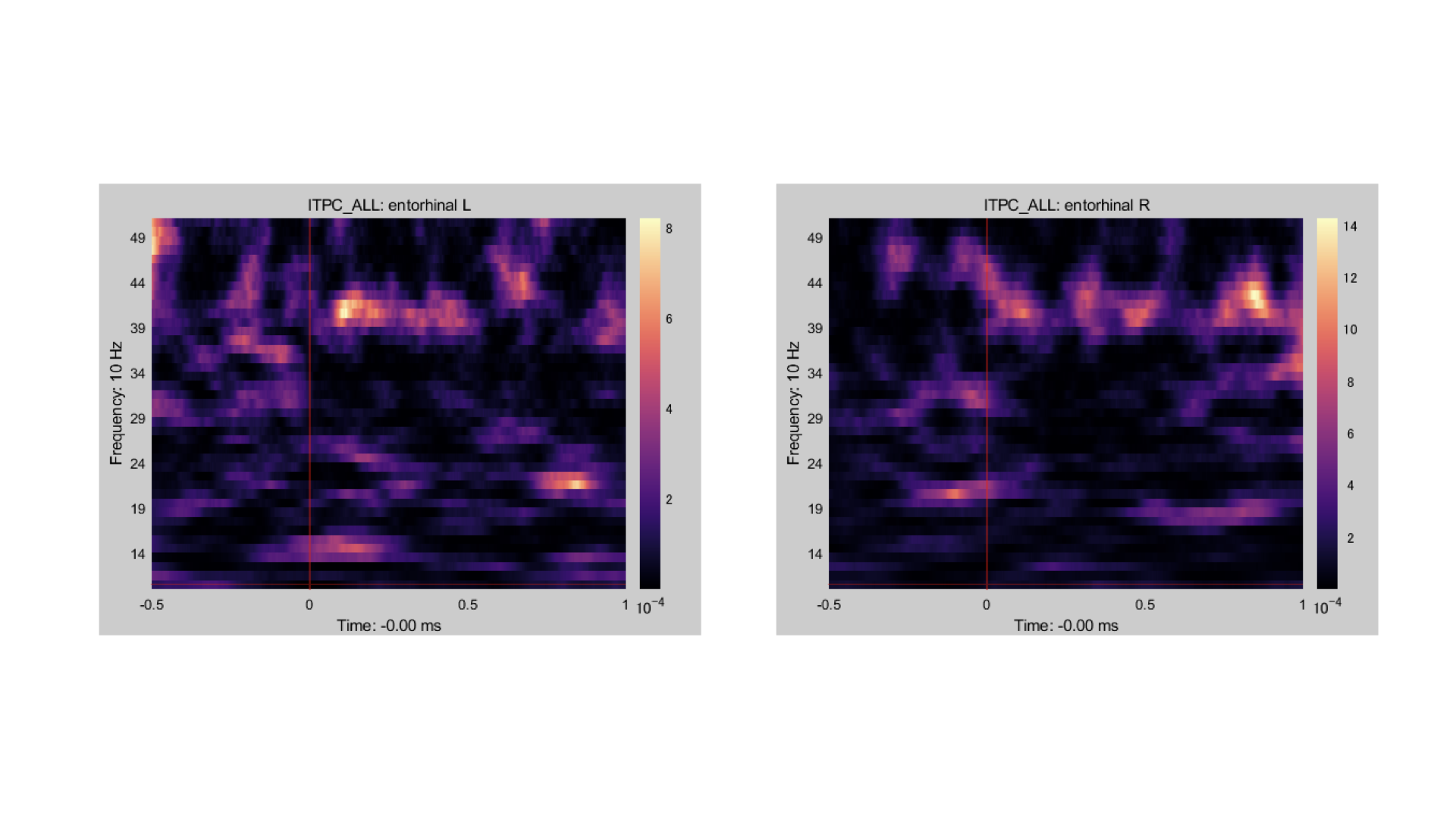

Supplement: Supplementary file 1 [file Image1.tiff]

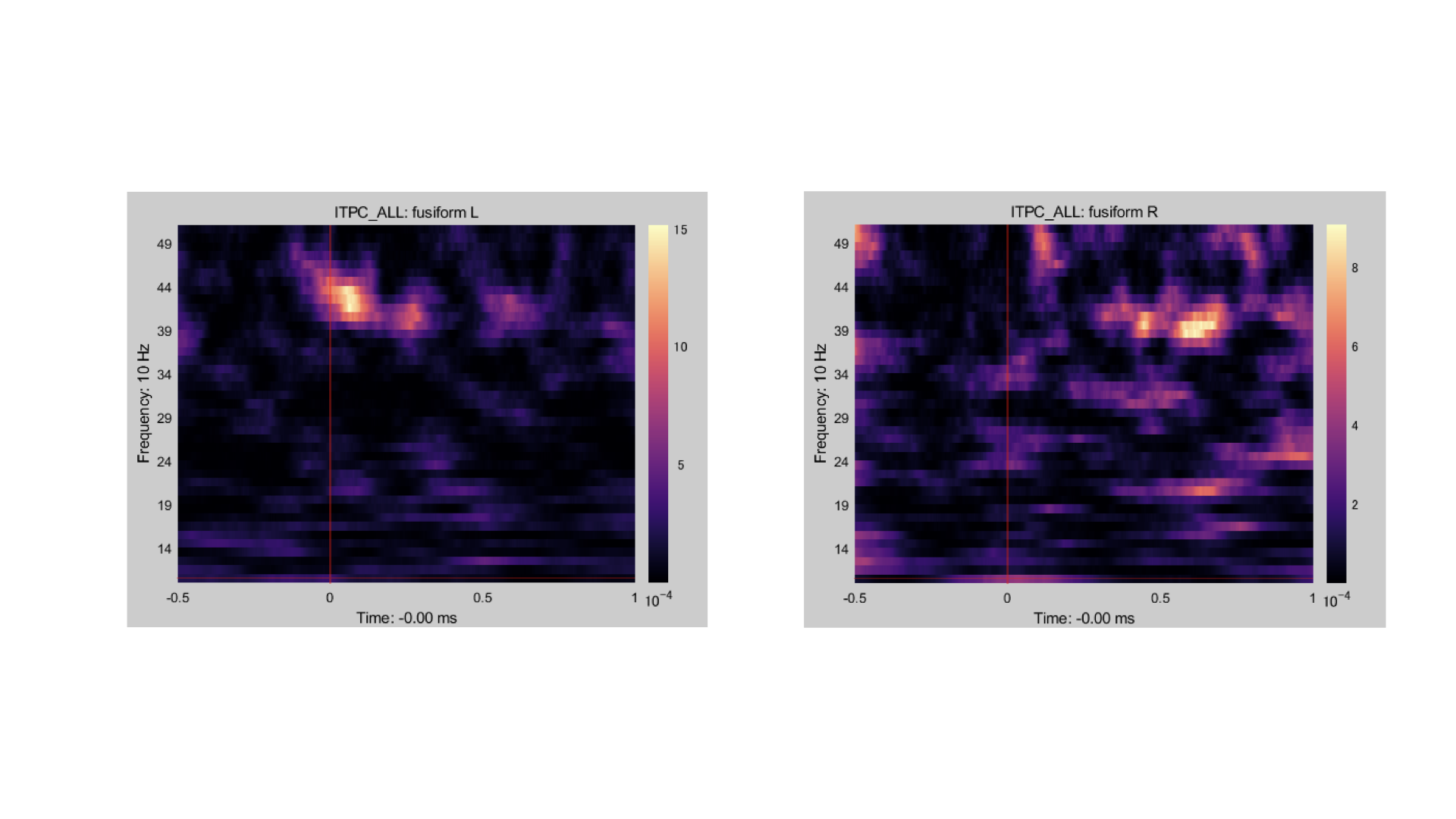

Supplement: Supplementary file 2 [file Image2.tiff]

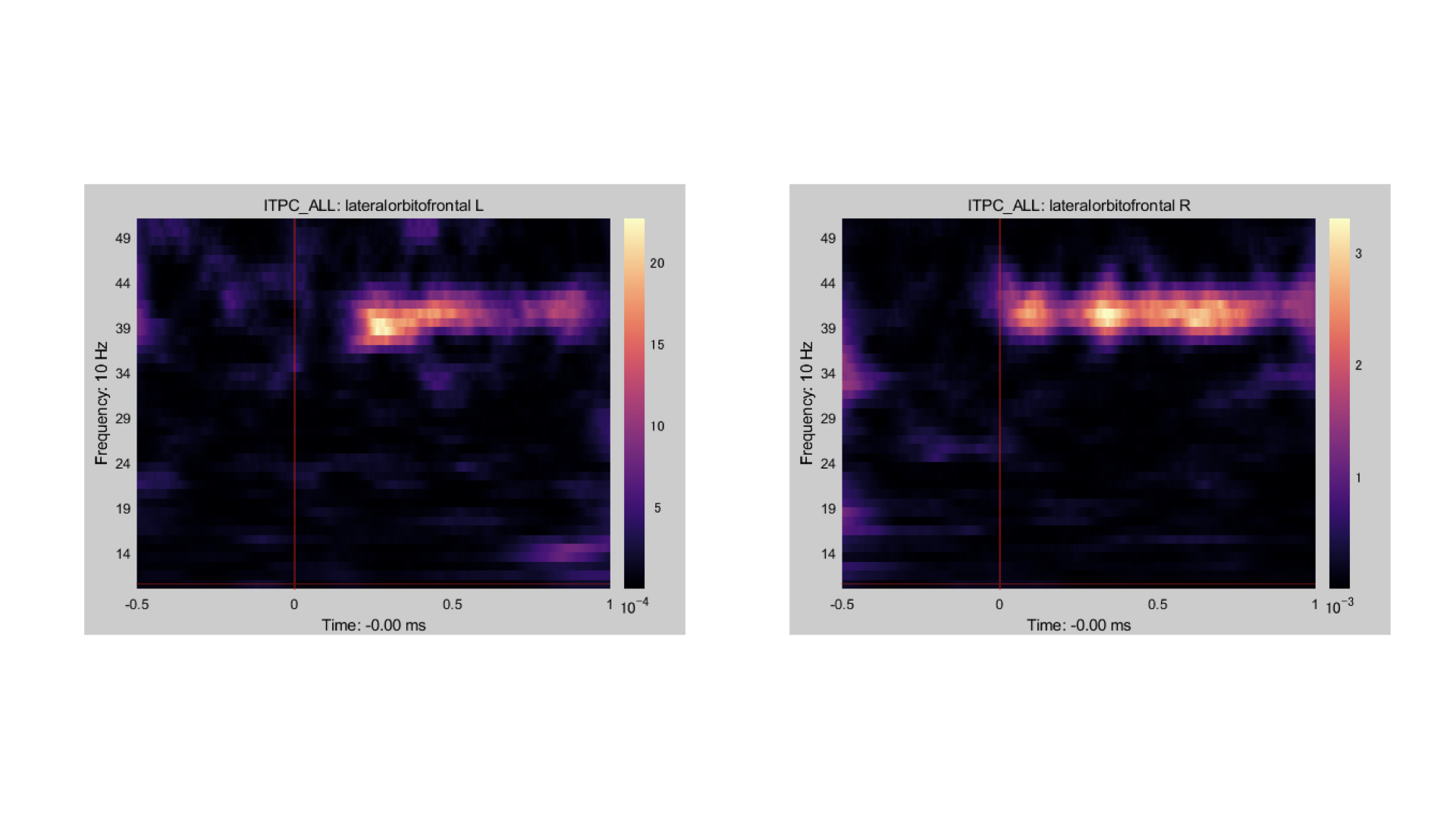

Supplement: Supplementary file 3 [file Image3.tiff]

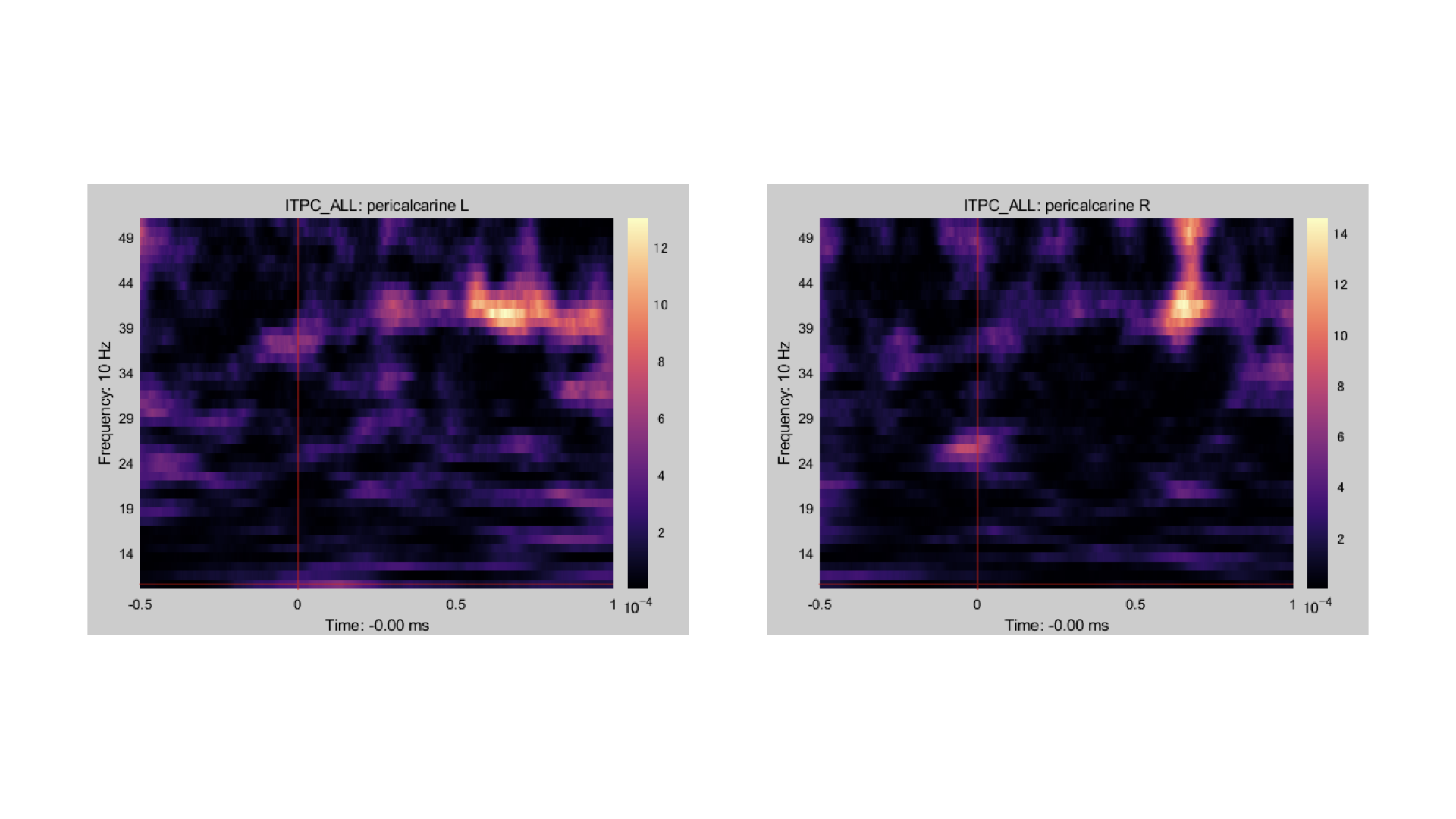

Supplement: Supplementary file 4 [file Image4.tiff]

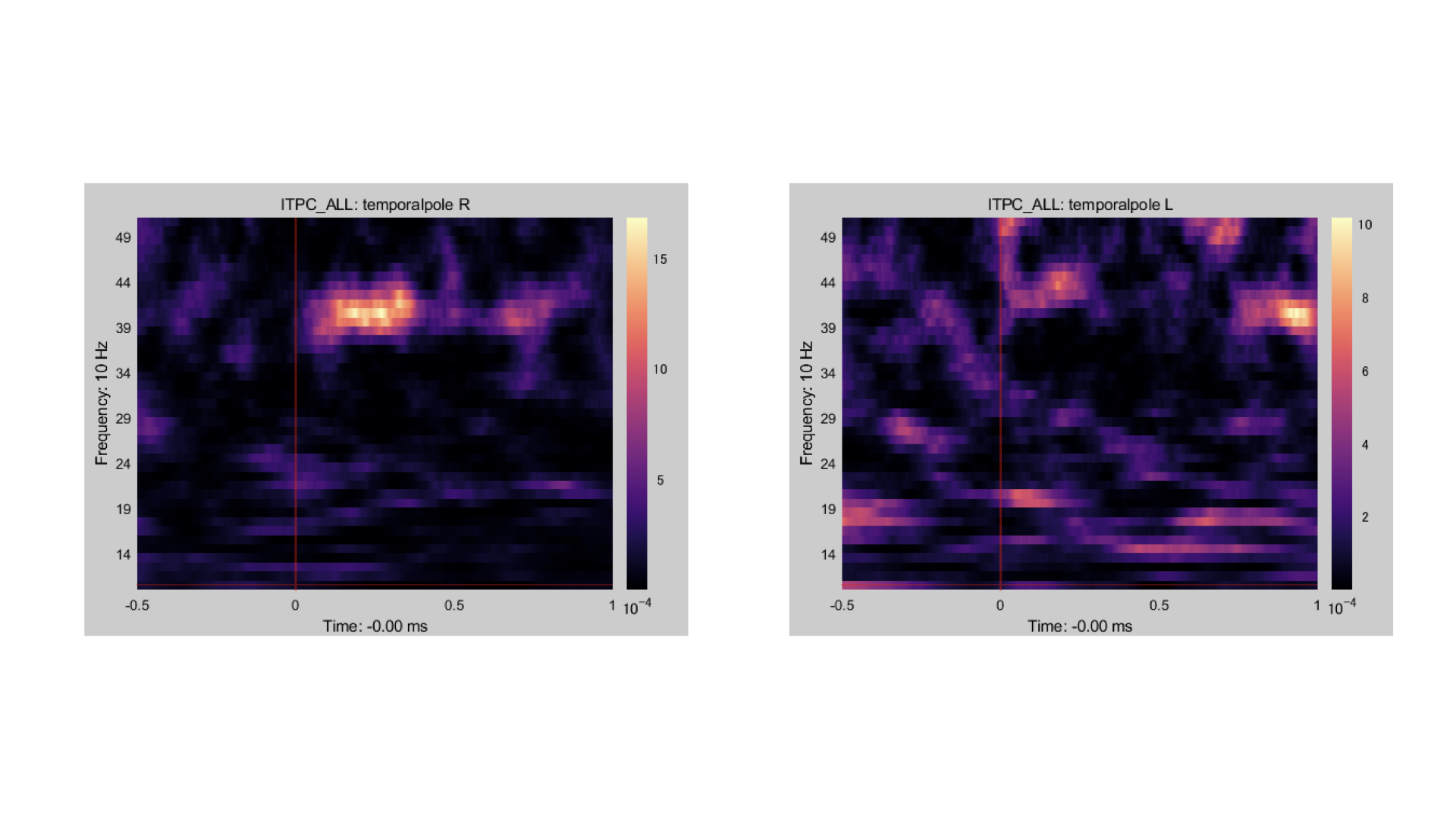

Supplement: Supplementary file 5 [file Image5.tiff]

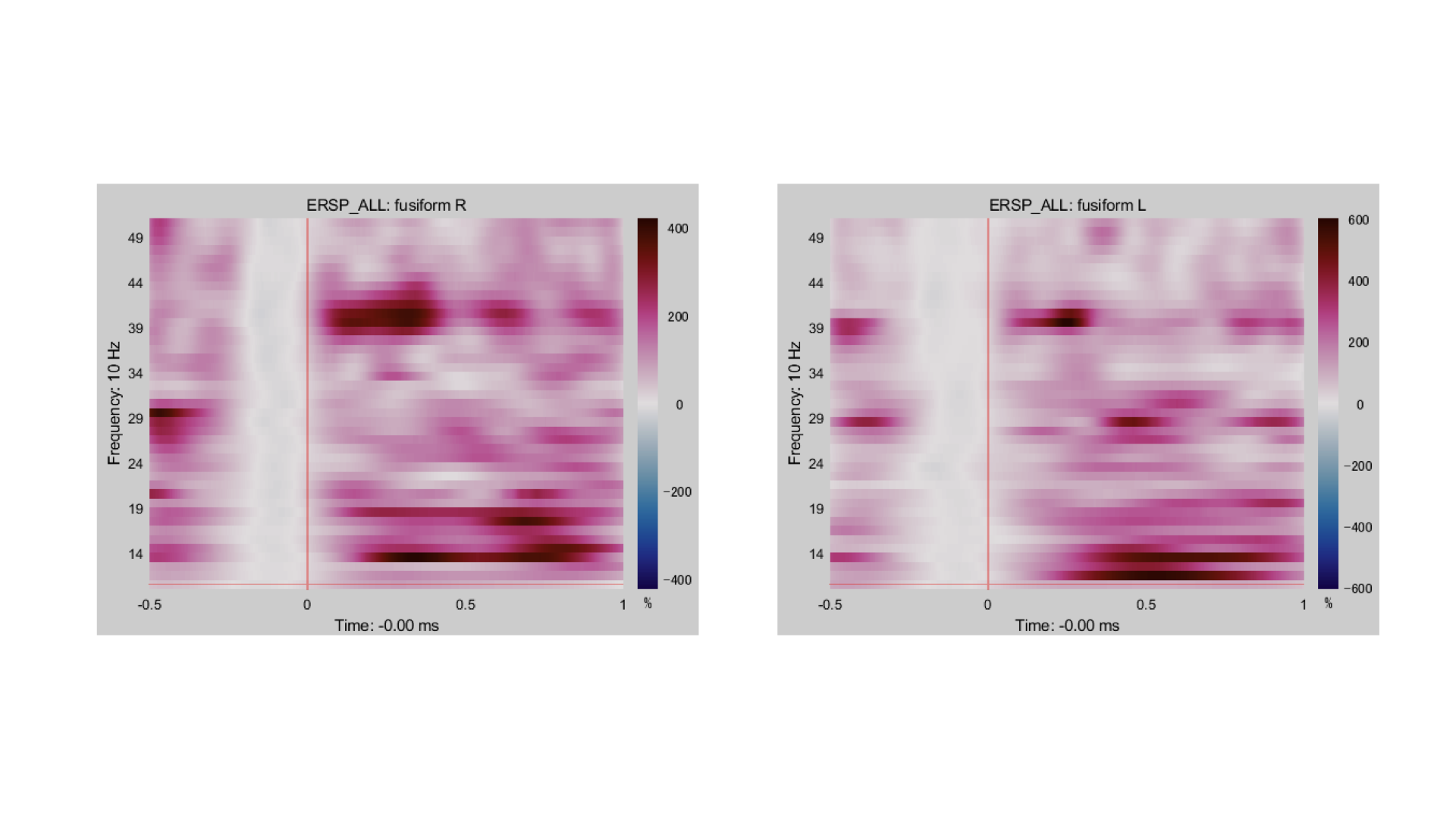

Supplement: Supplementary file 6 [file Image6.tiff]

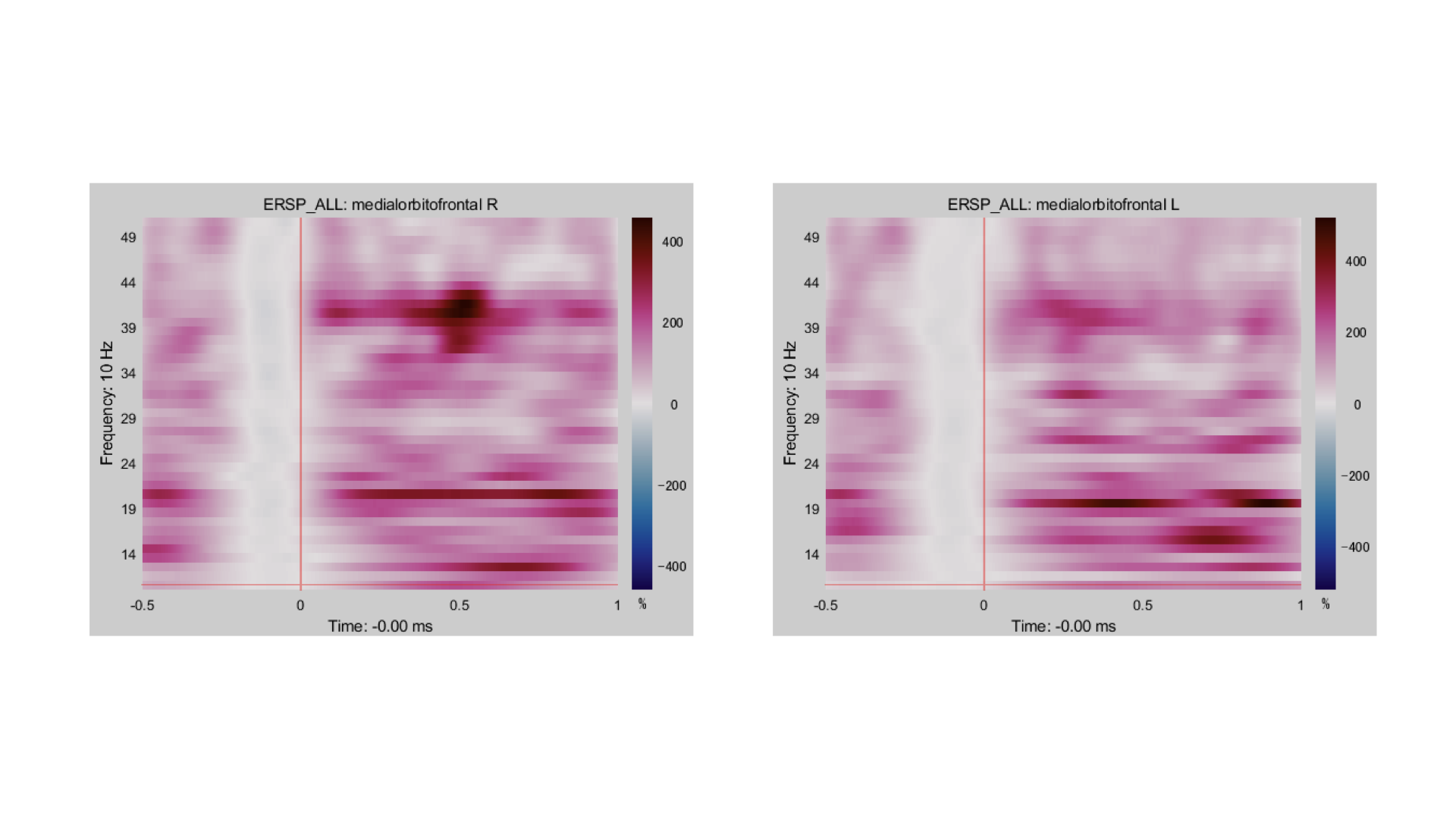

Supplement: Supplementary file 7 [file Image7.tiff]

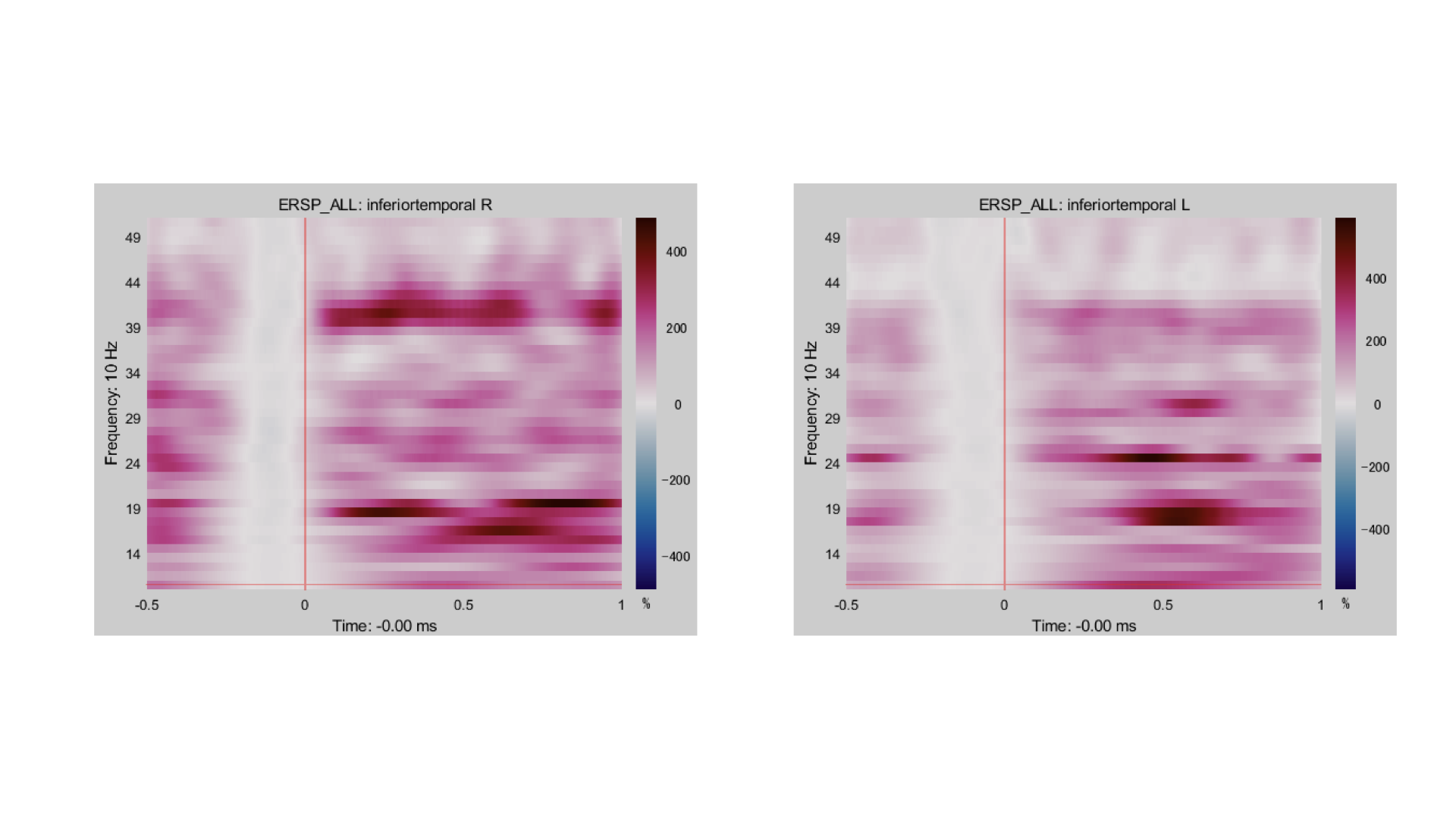

Supplement: Supplementary file 8 [file Image8.tiff]

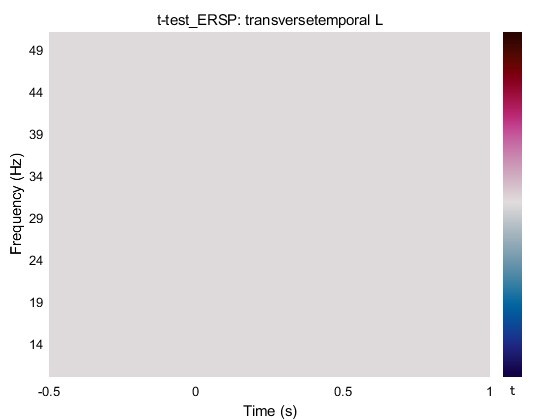

Supplement: Supplementary file 9 [file Image9.jpg]

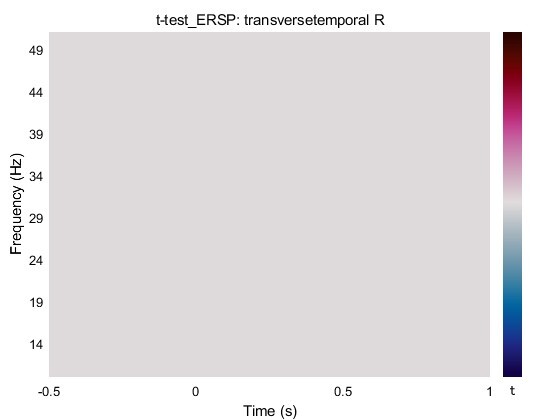

Supplement: Supplementary file 10 [file Image10.jpg]

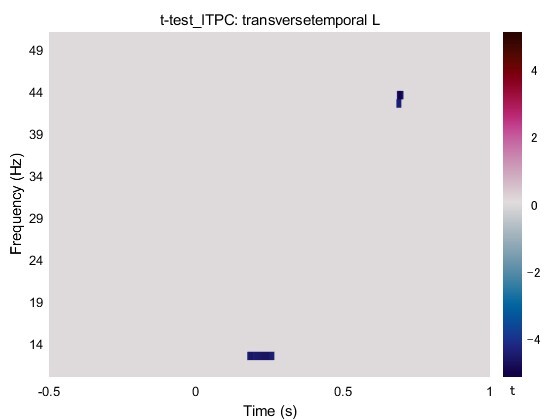

Supplement: Supplementary file 11 [file Image11.jpg]

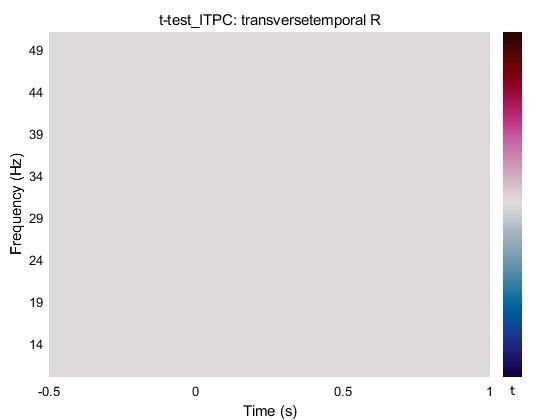

Supplement: Supplementary file 12 [file Image12.jpg]
