# Supplementary material for: Functional brain network organization during the 40-Hz auditory steady-state response in children with and without autism spectrum disorder
Source: Front Psychiatry. 2026 Jun 9;17:1804124. doi: 10.3389/fpsyt.2026.1804124 (PMC13288207; doi:10.3389/fpsyt.2026.1804124)
Supplement: Supplementary file 20 [file Table8.docx]

Supplementary Material

# Supplementary Data

**Supplementary Analysis 1. Group comparison of conventional ASSR measures in the bilateral transverse temporal gyri**

To supplement the descriptive validation of the 40-Hz ASSR paradigm shown in the main text, we conducted an additional group comparison of conventional ASSR measures in the bilateral transverse temporal gyri. For each participant, event-related spectral perturbation (ERSP) and inter-trial phase coherence (ITPC) were computed in the left and right transverse temporal gyri using the same source-level time–frequency analysis procedure described in the main Methods section.

Group differences between the ASD and TD groups were evaluated at each time–frequency bin using Welch’s t-tests. This yielded statistical maps across time, frequency, and hemisphere for ERSP and ITPC. For ERSP and ITPC separately, the resulting p-values were corrected for multiple comparisons using the false discovery rate (FDR) across all tested time–frequency bins, following the general approach used in our previous study employing the same stimulation paradigm in a similar age range (29).

The resulting FDR-corrected statistical maps are shown in Supplementary Figures S9–S12. No FDR-corrected group differences were observed for ERSP in either hemisphere. For ITPC, only a small number of isolated time–frequency bins in the left transverse temporal gyrus survived FDR correction, without forming a coherent sustained 40-Hz pattern. Accordingly, these findings were not interpreted as evidence of a robust group difference in conventional ASSR measures.

**Supplementary Table S1. Descriptive summary of source-level extra-auditory 40-Hz ASSR-related activity across cortical regions in the full sample.**

Source-level inter-trial phase coherence (ITPC) and event-related spectral perturbation (ERSP) were visually inspected across all cortical regions in the Desikan–Killiany atlas. For each region, 40-Hz ASSR-related activity outside the bilateral transverse temporal gyri was rated as absent, present, or prominent. These ratings were used for descriptive summarization only and were not intended as inferential statistical results.

**Supplementary Table S2. Threshold–sensitivity analysis for clustering coefficient (CC).**

Results of linear regression analyses examining diagnostic effects on CC across a range of proportional thresholds surrounding the main analysis threshold. For each threshold, models were adjusted for age in months, sex, and mental processing abilities, as measured by the K-ABC, and estimated using heteroscedasticity-robust standard errors.

**Supplementary Table S3. Threshold–sensitivity analysis for characteristic path length (PL).**

Results of linear regression analyses examining diagnostic effects on characteristic path length (PL) across a range of proportional thresholds surrounding the main analysis threshold. For each threshold, models were adjusted for age in months, sex, and mental processing abilities, as measured by the K-ABC, and estimated using heteroscedasticity-robust standard errors.

**Supplementary Table S4. Threshold–sensitivity analysis for small-worldness (SW).**

Results of linear regression analyses examining diagnostic effects on small-worldness (SW) across a range of proportional thresholds surrounding the main analysis threshold. For each threshold, models were adjusted for age in months, sex, and mental processing abilities, as measured by the K-ABC, and estimated using heteroscedasticity-robust standard errors.

**Supplementary Table S5. Exploratory threshold–sensitivity analysis for the association between characteristic path length (PL) and SRS total T-scores.**

Results of exploratory linear regression analyses examining the association of characteristic path length (PL), diagnosis (ASD vs. TD), and their interaction with SRS total T-scores across a range of proportional thresholds surrounding the main analysis threshold. For each threshold, models were adjusted for age in months, sex, and mental processing abilities, as measured by the K-ABC, and estimated using heteroscedasticity-robust standard errors.

**Supplementary Table S6. Sensitivity analysis including diagnosis × mental processing ability interaction terms for graph-theoretical measures at the main analysis threshold (κ = 0.20).**

Results of linear regression models for clustering coefficient (CC), small-worldness (SW), and characteristic path length (PL) including diagnosis (ASD vs. TD), mental processing ability as measured by the K-ABC, their interaction term, age in months, and sex as predictors. Models were estimated using heteroscedasticity-robust standard errors.

**Supplementary Table S7. Sensitivity analysis of the characteristic path length (PL)–SRS model including a diagnosis × mental processing ability interaction term at the main analysis threshold (κ = 0.20).**

Results of the linear regression model predicting SRS total T-scores from characteristic path length (PL), diagnosis (ASD vs. TD), the PL × diagnosis interaction, mental processing ability as measured by the K-ABC, the diagnosis × mental processing ability interaction, age in months, and sex. The model was estimated using heteroscedasticity-robust standard errors.

**Supplementary Figures S1–S5. Representative ITPC time–frequency plots in extra-auditory cortical regions.**

Grand-averaged time–frequency representations of inter-trial phase coherence (ITPC) in the entorhinal cortex (Figure S1), fusiform gyrus (Figure S2), lateral orbitofrontal cortex (Figure S3), pericalcarine cortex (Figure S4), and temporal pole (Figure S5) in the full sample. In each region, increased phase-locking activity is observable around 40 Hz during auditory stimulation.

**Supplementary Figures S6–S8. Representative ERSP time–frequency plots in extra-auditory cortical regions.**

Grand-averaged time–frequency representations of event-related spectral perturbation (ERSP) in the fusiform gyrus (Figure S6), medial orbitofrontal cortex (Figure S7), and inferior temporal cortex (Figure S8) in the full sample. In each region, increased spectral power around 40 Hz is observable during auditory stimulation.

**Supplementary Figure S9. FDR-corrected group comparison map for ERSP in the left transverse temporal gyrus.**

Time–frequency t-map showing differences in event-related spectral perturbation (ERSP) between the ASD and TD groups in the left transverse temporal gyrus. Welch’s t-tests were performed at each time–frequency bin, and resulting p-values were corrected using the false discovery rate across all tested time–frequency bins and both hemispheres for ERSP.

**Supplementary Figure S10. FDR-corrected group comparison map for ERSP in the right transverse temporal gyrus.**

Time–frequency t-map showing differences in event-related spectral perturbation (ERSP) between the ASD and TD groups in the right transverse temporal gyrus. Welch’s t-tests were performed at each time–frequency bin, and resulting p-values were corrected using the false discovery rate across all tested time–frequency bins and both hemispheres for ERSP.

**Supplementary Figure S11. FDR-corrected group comparison map for ITPC in the left transverse temporal gyrus.**

Time–frequency t-map showing differences in inter-trial phase coherence (ITPC) between the ASD and TD groups in the left transverse temporal gyrus. Welch’s t-tests were performed at each time–frequency bin, and resulting p-values were corrected using the false discovery rate across all tested time–frequency bins and both hemispheres for ITPC.

**Supplementary Figure S12. FDR-corrected group comparison map for ITPC in the right transverse temporal gyrus.**

Time–frequency t-map showing differences in inter-trial phase coherence (ITPC) between the ASD and TD groups in the right transverse temporal gyrus. Welch’s t-tests were performed at each time–frequency bin, and resulting p-values were corrected using the false discovery rate across all tested time–frequency bins and both hemispheres for ITPC.
